# Decitabine-Vorinostat combination treatment in acute myeloid leukemia activates pathways with potential for novel triple therapy

**DOI:** 10.18632/oncotarget.18009

**Published:** 2017-05-19

**Authors:** Christine S. Young, Kathryn M. Clarke, Laura M. Kettyle, Alexander Thompson, Ken I. Mills

**Affiliations:** ^1^ Blood Cancer Research Group, Centre for Cancer Research and Cell Biology, Queen's University, Belfast, United Kingdom; ^2^ MRC Human Genetics Unit, Institute of Genetics and Molecular Medicine, University of Edinburgh, Western General Hospital, Edinburgh, United Kingdom; ^3^ Department of Haematology, Addenbrooke's Hospital, Cambridge, United Kingdom; ^4^ Haematopoietic Stem Cell Biology Laboratory, MRC Molecular Haematology Unit, Weatherall Institute of Molecular Medicine, John Radcliffe Hospital, Oxford, United Kingdom; ^5^ Division of Cancer and Stem Cells, Centre for Biomolecular Sciences, University of Nottingham, Nottingham, United Kingdom

**Keywords:** epigenetic combination therapies, AXL receptor tyrosine kinase, acute myeloid leukemia, HDAC inhibitors, DNMT inhibitors

## Abstract

Despite advancements in cancer therapeutics, acute myeloid leukemia patients over 60 years old have a 5-year survival rate of less than 8%. In an attempt to improve this, epigenetic modifying agents have been combined as therapies in clinical studies. In particular combinations with Decitabine and Vorinostat have had varying degrees of efficacy. This study therefore aimed to understand the underlying molecular mechanisms of these agents to identify potential rational epi-sensitized combinations.

Combined Decitabine-Vorinostat treatment synergistically decreased cell proliferation, induced apoptosis, enhanced acetylation of histones and further decreased DNMT1 protein with HL-60 cells showing a greater sensitivity to the combined treatment than OCI-AML3. Combination therapy led to reprogramming of unique target genes including *AXL*, a receptor tyrosine kinase associated with cell survival and a poor prognosis in AML, which was significantly upregulated following treatment. Therefore targeting AXL following epi-sensitization with Decitabine and Vorinostat may be a suitable triple combination. To test this, cells were treated with a novel triple combination therapy including BGB324, an AXL specific inhibitor. Triple combination increased the sensitivity of OCI-AML3 cells to Decitabine and Vorinostat as shown through viability assays and significantly extended the survival of mice transplanted with pretreated OCI-AML3 cells, while bioluminescence imaging showed the decrease in disease burden following triple combination treatment.

Further investigation is required to optimize this triple combination, however, these results suggest that AXL is a potential marker of response to Decitabine-Vorinostat combination treatment and offers a new avenue of epigenetic combination therapies for acute myeloid leukemia.

## INTRODUCTION

Acute myeloid leukemia (AML) is the most common form of adult leukemia with disease heterogeneity contributing significantly to variability in patient outcomes. Although survival rates of younger patients have increased in recent years, there has been little improvement for elderly patients (> 60 yrs. old) who still have a 5 year survival rate of less than 8% [1]. Although subclassification of AML has greatly improved during this time, little progression has been made in developing effective targeted therapies for the treatment of AML. The current standard of care regimen of induction (daunorubicin and cytarabine) followed by consolidation is only slightly amended from original protocols [2].

Aberrant epigenetic modifications including methylation (DNA and histone) and acetylation (histone) are key contributors to leukemia initiation and maintenance [3]. A number of epigenetic modifying therapies (EMTs) have shown promise in reversing these abnormal patterns and reinstating an epigenetic landscape that resembles a more normal state. As early as 1999, DNA methyltransferase inhibitors (DNMTi) and histone deacetylase inhibitors (HDACi) were shown to have synergistic antitumor activities through the re-expression of silenced genes [4].

DNMTi 5-aza-2′-deoxycytidine (Decitabine) and HDACi Vorinostat (SAHA), have been examined as single agent therapies for the treatment of AML [5, 6]. In an attempt to improve efficacy, various combination therapies have been tested, many of which have been extended to clinical trial [7, 8]. Significant variability in the degree of efficacy has been reported for Decitabine treatment in combination with Vorinostat from a response rate of 14% for sequential treatment to 46% for concurrent treatment in AML and myelodysplastic syndrome [9].

The ability of these EMTs to reverse aberrant epigenetic marks and re-sensitize or ‘epi-sensitize’ some tumors to treatments to which they had acquired resistance, [10–12] makes them an attractive therapeutic avenue for further development. Although this re-sensitizing effect is seen in some instances, this is not always the case as a number of studies have reported [13, 14]. None-the-less, their perceived reduced toxicities make it an attractive option for patients unfit for the current more toxic regimens [15, 16]. While Decitabine and Vorinostat have shown synergistic *in vitro* anti-tumor effects in both hematological malignancies and various solid tumors, [17–21] the molecular mechanisms are not fully understood and require further consideration. This study aimed to investigate the synergistic affects of Decitabine-Vorinostat (DV) combination treatment in AML subtypes, identify candidate mechanisms associated with disease subtype-specific sensitivity and identify pathways that could be targeted following epi-sensitization with DV treatment. Combination gene expression signatures were obtained from AML subtypes and the receptor tyrosine kinase *AXL* was identified as a sensitivity-associated candidate and potential target for triple combination therapy.

## RESULTS

### The sequential addition of Vorinostat to Decitabine primed cells results in a synergistic reduction in cell viability

**Figure 1:**
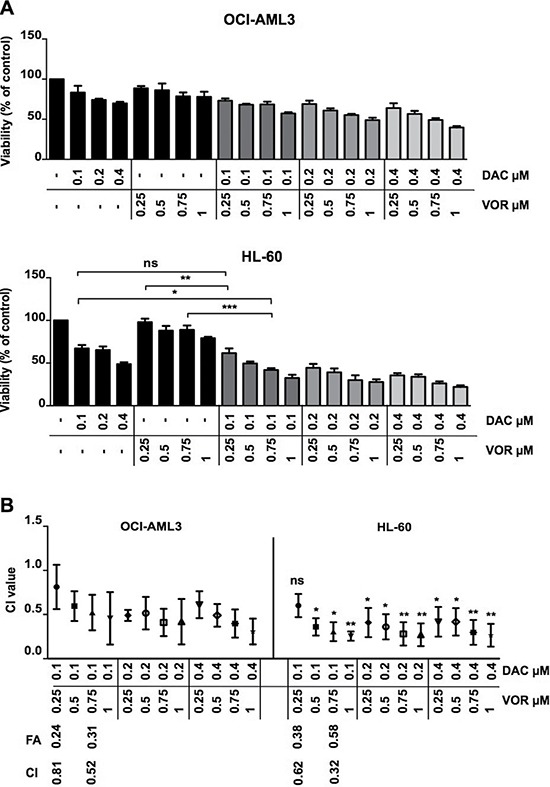
Sequential Decitabine and Vorinostat combination treatment synergistically inhibits AML cell viability (**A**) OCI-AML3 (Top) and HL-60 (Bottom) cells were treated with DAC (0.1 μM, 0.2 μM and 0.4 μM), VOR (0.25 μM, 0.5 μM, 0.75 μM and 1 μM) and all DV combination doses in a sequential manner for a total of 72 hours. Cell viability was measured using a CellTitre-Glo^®^ assay. (**B**) Viability percentage was used to calculate the combination index by Calcusyn software. The combination index for each combination is depicted in the graph. The Fraction affected (FA) by treatments and combination index (CI) values for candidate doses taken forward are highlighted in this figure. Data represent mean ± SEM; *n* = 3 (***= *p* < 0.001; **= *p* < 0.01; *= *p* < 0.05).

Single agent treatment with Decitabine or Vorinostat resulted in reduced viability of AML cell lines. IC50 concentrations for OCI-AML3 and HL-60 cells were in the low micro-molar range for each agent (Supplementary Figure 1). To evaluate potential synergy between these EMTs, cell lines were treated with a range of dose combinations (concurrent and sequential) and the combination index (CI) was calculated using Calcusyn software as described in the methods. The doses chosen for combination studies (DAC 0.1-0.4 μM and VOR 0.25-1 μM) were shown previously in our lab to avoid large-scale cytotoxic cell kill. Sequential dose combinations reduced cell viability (Figure 1A) further than single agent treatments, achieving a high degree of synergy at lower concentrations of each agent while the concurrent regimen required higher doses to achieve only a minor synergistic effect as determined by the CI value (Supplementary Figure 2). The degree of synergy was more notable in HL-60 compared to OCI-AML3 cells. Due to a greater degree of synergy found using sequential dosing compared to concurrent dosing, this schedule was taken forward for further analysis. The combination index for the four dose combinations taken forward are highlighted in Figure 1B. Those taken forward provided a combination with a low degree and high degree of synergy for comparison. All combination index values for sequential doses tested are outline in Supplementary Table 1.

### Decitabine/Vorinostat combination treatment induced apoptotic cell death

**Figure 2:**
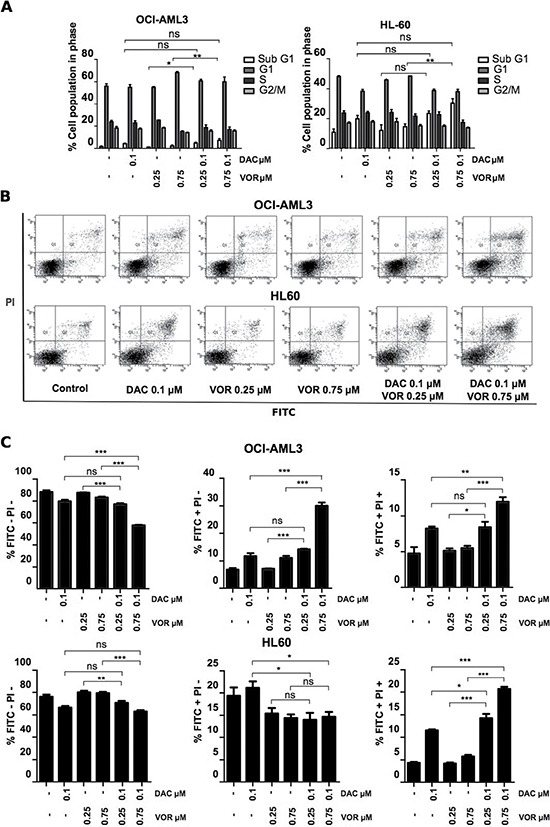
Combined Decitabine and Vorinostat treatment induces an increase in apoptosis in AML cell lines OCI-AML3 and HL-60 cells were treated with 0.1 μM DAC, 0.25 μM and 0.75 μM VOR and both DV combination doses in a sequential manner. Cells were harvested and the cell cycle profile of (**A**) OCI-AML3 (left) and HL-60 (right) cells following treatment was analysed by FACS analysis. Annexin V PI staining and the percentage induction of early and late apoptotic cell populations were quantified by FACS analysis in OCI-AML3 and HL-60 cells. Results are depicted as dot plots (**B**) showing the migration from FITC-/PI- (live cells) to FITC+/PI- (early apoptotic) and FITC+/PI+ (late apoptotic) populations and quantified as a percentage (**C**) for each staining condition. Data represent mean± SEM; *n* = 3 (***= *p* < 0.001; **= *p* < 0.01; *= *p* < 0.05).

Cell cycle profiling highlighted the difference in cell sensitivity between the OCI-AML3 and HL-60 cell lines. The most notable effects were an increase (~10%) in the G1 phase following single agent Vorinostat treatment in the OCI-AML3 cells and an increase in the SubG1 population from 1% to 7% and 11% to 30% in the OCI-AML3 and HL-60 cells respectively following higher dose DV combination treatment (Figure 2A).

Annexin V and PI staining confirmed a significant increase in apoptotic cell death following DV combination treatment compared to the control and single treatments. Higher dose DV treatment significantly reduced the live cell population, increased the early apoptotic population (FITC+/PI-) by 23% in the OCI-AML3 cells, decreased it by 5% in the HL-60 cells and further increased the late apoptotic population (FITC+/PI+) by 7% and 16% in OCI-AML3 and HL-60 cells respectively relative to the control (Figure 2B–2C). This highlights a greater sensitivity in the HL-60 cells to DV combination treatment.

### The addition of Vorinostat to decitabine primed cells enhanced caspases activity

**Figure 3:**
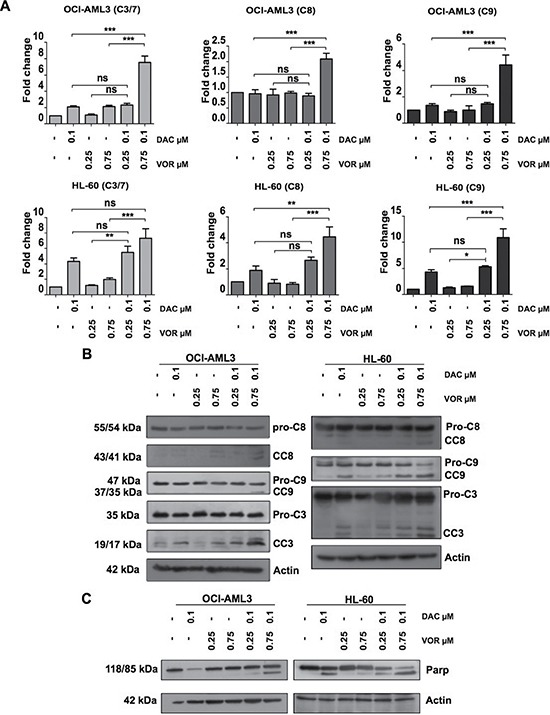
Combined Decitabine and Vorinostat treatment induces caspase activation and Parp cleavage in AML cell lines OCI-AML3 and HL-60 cells were treated with 0.1 μM DAC, 0.25 μM and 0.75 μM VOR and both DV combination doses in a sequential manner. (**A**) Caspase activities were analysed in OCI-AML3 (top panel) and HL-60 (bottom panel) cells using Caspase 3/7 (left), 8 (centre) and 9 (right) Glo assays. Relative light units were normalised to the percentage of viable cells. (**B**) Caspase cleavage and (**C**) Parp cleavage of OCI-AML3 (left panel) and HL-60 (right panel) cells following each treatment were assessed by western blot analysis of total cell lysate. Actin is used as a loading control. Data represent mean± SEM; *n* = 3 (***= *p* < 0.001; **= *p* < 0.01; *= *p* < 0.05).

To determine whether the increase in apoptosis was due to the activation of caspase enzymes, the level of caspase cleavage following single agent treatment and combination treatment were assessed. While the higher dose of Vorinostat alone had a minimal effect on caspase 3/7 activity in both cell lines (~1 fold), Decitabine alone enhanced caspase 9 (1 fold) and caspase 3/7 (2 fold) activity in OCI-AML3 cells, while increasing caspase 8 (1 fold), caspase 9 (4 fold) and caspase 3/7 (4 fold) activity in HL-60 cells (Figure 3A). High dose DV combination treatment synergistically increased the levels of caspase 8, 9 and 3/7 activity further; 7, 2 and 4 fold respectively in OCI-AML3 cells and 4, 10 and 7 fold respectively in HL-60 cells (Figure 3A). Immunoblotting analysis further confirmed this increase in caspase activity through the cleavage of caspase proteins from their inactive to active forms (Figure 3B). Supporting the findings from Figure 3A, immunoblotting of lysates treated with Decitabine alone also showed increased levels of cleaved caspase 3. Caspase 3, 8 and 9 cleavage is also apparent following the higher dose of DV treatment in both cell lines. In addition to caspase cleavage, PARP, a downstream target of caspase 3 was cleaved upon Decitabine treatment in HL-60 cells and DV treatment in both cell lines, (Figure 3C) further supporting a role for apoptotic signalling following combination treatment.

### Combination treatment enhanced histone H4 acetylation and further decrease DNMT1 protein

**Figure 4:**
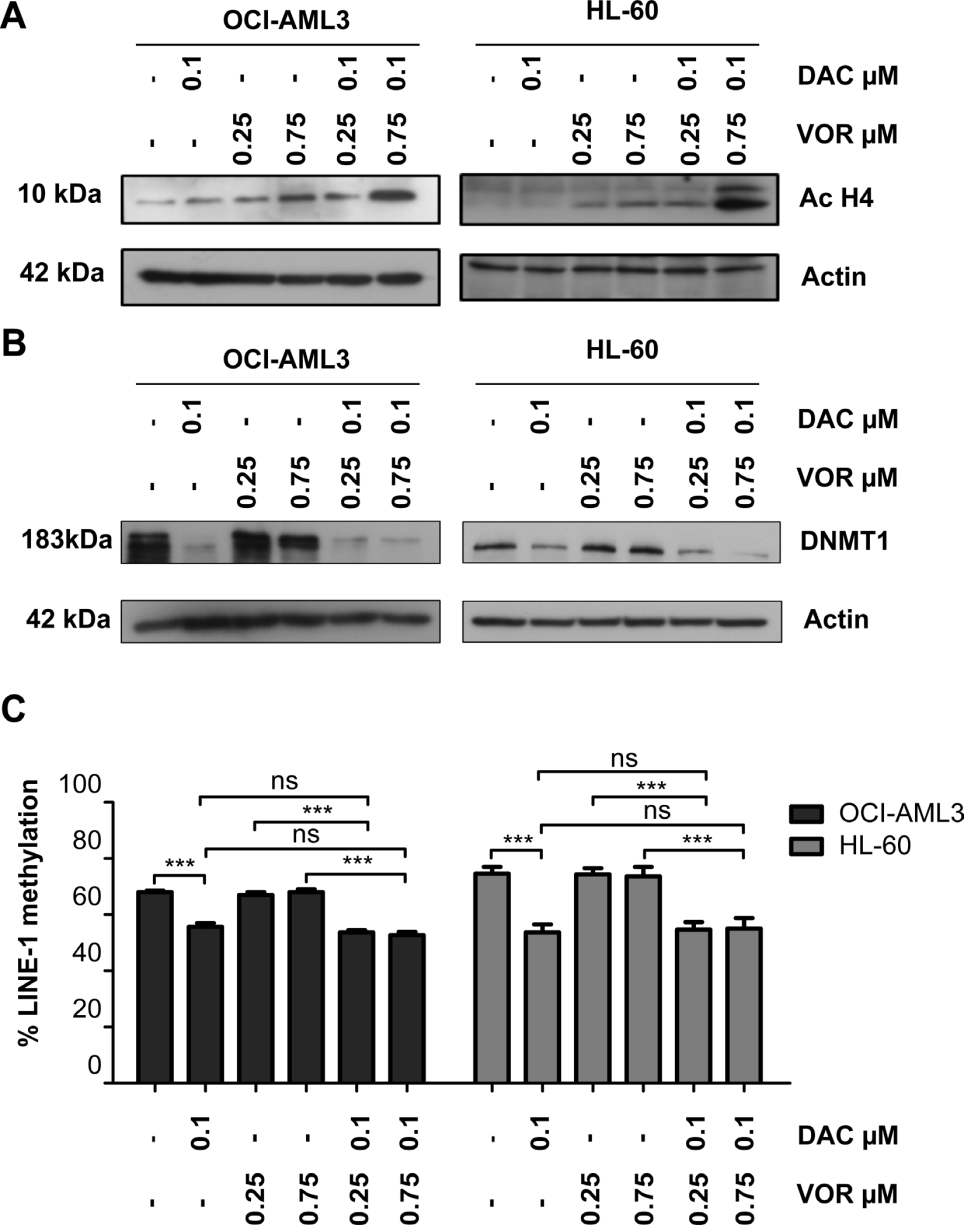
Combined Decitabine and Vorinostat treatment enhances histone acetylation and further decreased DNMT1 expression in AML cell lines OCI-AML3 and HL-60 cells were treated with 0.1 μM DAC, 0.25 μM and 0.75 μM VOR and both DV combination doses in a sequential manner. Western blot analysis of total cell lysates was used to assess the levels of (**A**) histone H4 acetylation and (**B**) DNMT1 protein in OCI-AML3 (left) and HL-60 (right) cells following treatment. Actin is used as a loading control. Matching samples were taken for methylation analysis. (**C**) DNA was extracted, bisulfite converted and CpG methylation of the human LINE-1 sequence was quantified by pyrosequencing to estimate levels of global DNA methylation following each treatment in OCI-AML3 and HL-60 cells. Data represent mean± SEM; *n* = 3 (***= *p* < 0.001; **= *p* < 0.01; *= *p* < 0.05).

As expected, Vorinostat treatment alone resulted in histone H4 acetylation in a dose dependent manner (Supplementary Figure 1B). Western blotting demonstrated an enhanced level of histone H4 acetylation following combination treatments, particularly at higher doses compared to Vorinostat alone (Figure 4A).

We next assessed the impact of combined treatment on DNMT1 protein levels and activity. Decitabine alone decreased DNMT1 protein levels and LINE-1 CpG methylation in a dose dependent manner (Supplementary Figure 1B–1C). No further decrease in DNMT1 protein levels were observed in OCI-AML3 cells with the addition of Vorinostat (Figure 4B); probably as a result of the already significantly diminished levels with Decitabine alone. Interestingly, the higher dose combination further decreased the DNMT1 protein level in HL-60 cells (Figure 4B); but did not result in enhanced LINE-1 CpG demethylation (Figure 4C).

### Combination treatment generates a unique gene expression signature

To identify the subsequent gene expression changes induced as a result of altered epigenetic status, microarray analysis was performed on OCI-AML3 cells treated with single agents or the DV combination while qRT-PCR validation of candidate genes identified from this analysis was performed on the HL-60 treatments.

**Figure 5:**
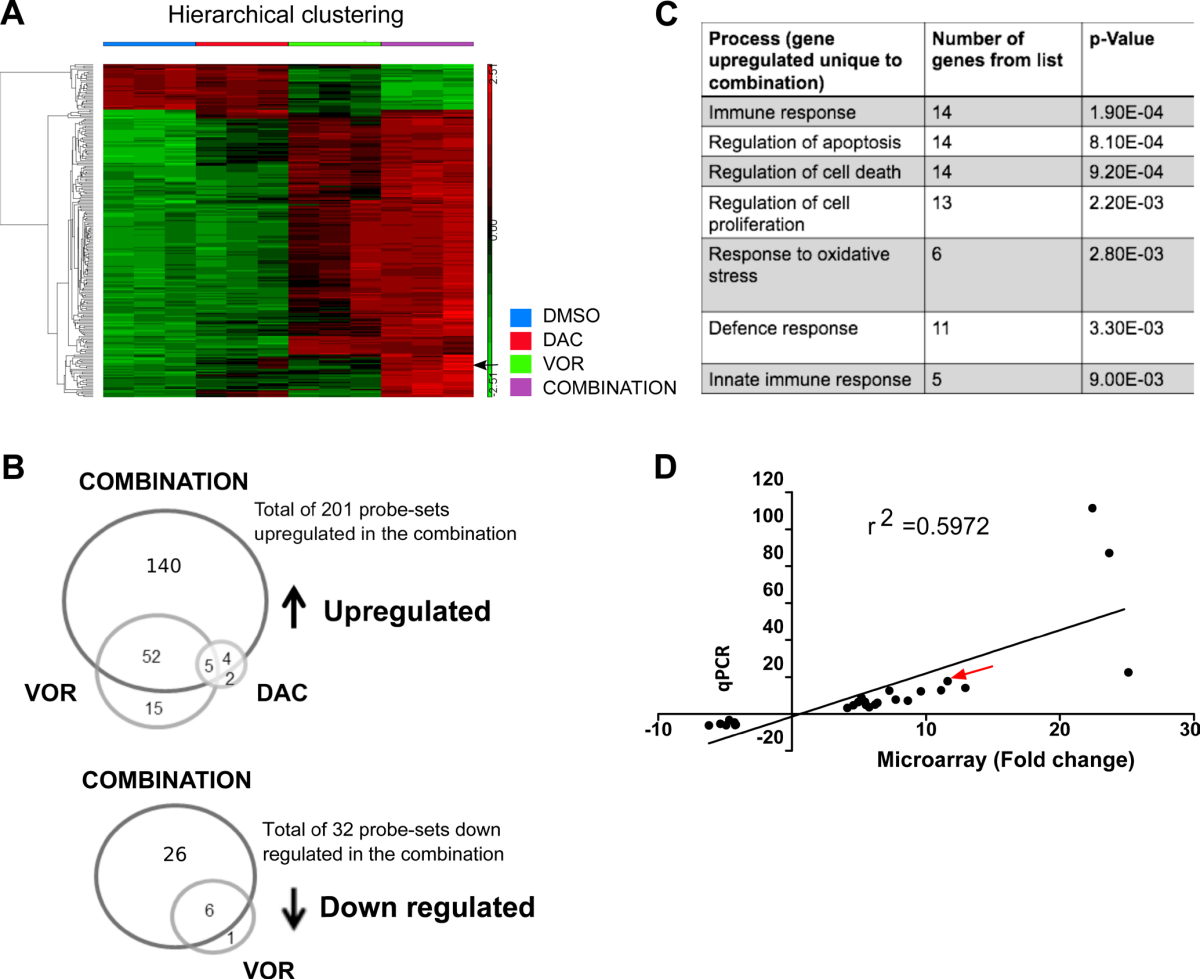
Combined Decitabine and Vorinostat treatment induces unique gene expression changes in AML cell lines OCI-AML3 cells were treated with 0.1 μM DAC, 0.75 μM VOR and the DV combination in a sequential manner. RNA was extracted, processed to cRNA and hybridised to Affymetrix U133 Plus 2 array chips (*n* = 3). Following processing of array chips, CEL files were uploaded to Partek Genomic Suite and an ANOVA analysis performed to detect differentially expressed probe-sets between the control and each treatment group. (**A**) Hierarchical clustering performed on 233 probe-sets identified as differentially expressed between the control and the DV combination treatment (upregulated probe-sets = red; downregulated probe-sets = green). Black arrow indicates AXL gene expression (**B**) Differential probe-set lists were overlapped to identify probe-sets (genes) that were altered uniquely following DV combination treatment. (**C**) Probe-sets found to be uniquely altered following DV treatment were subjected to Partek gene ontology analysis identifying processes specifically enriched with this treatment. (**D**) A cohort of 27 genes (19 upregulated and 8 downregulated) uniquely altered following DV combination treatment were chosen for qRT-PCR validation. The Pearson correlation coefficient confirms correlation between the fold change obtained from the microarray data and qRT-PCR validation (*r*^2^ = 0.5972).

Only 11 probe-sets were significantly increased following Decitabine treatment alone while 79 probe-sets were significantly altered by Vorinostat (72 increased and 7 decreased) (Supplementary Table 2). In contrast, a total of 233 probe-sets had altered expression following DV combination treatment (201 increased and 32 decreased) (Figure 5A). Figure 5B uses Venn diagrams to show the number of probe-sets that were regulated (top panel - up-regulated or bottom panel - downregulated) by more than one treatment condition. This analysis also allowed us to identify a sub-category of probe-sets that were unique to each treatment group. We identified a total of 140 probe-sets upregulated and 26 downregulated (118 and 24 genes respectively) exclusively following combination treatment.

Partek gene ontology (GO) and the DAVID database [22, 23] analysis of the expression data identified immune response, regulation of cell death, regulation of cell proliferation and response to oxidative stress as highly enriched processes (Figure 5C). A subset of 27 genes were chosen at random from those listed in the enriched pathways highlighted in Figure 5C for validation by qRT-PCR. Analysis of this data showed that the expression levels detected by qRT-PCR (19 upregulated and 8 downregulated) correlated strongly with the microarray results (*r*^2^ = 0.5972; Figure 5D). The receptor tyrosine kinase *AXL* was identified as one of the most upregulated genes from both the microarray and validation studies and is highlighted by the arrow on the heat map in Figure 5A and on the linear regression analysis presented in Figure 5D. Interestingly, this gene is also involved in each of the most highly enriched processes listed in Figure 5C. On further investigation of the microarray data, a number of AXL associated/target genes were identified as being upregulated following DV treatment (Supplementary Figure 3A–3C). These included TWIST and SOCS1 but not SOCS3 all of which aim to suppress pro-inflammatory signals. In addition, an increase in phospho-ERK1/2, a known downstream AXL signaling molecule was observed following DV treatment in OCI-AML3 cells (Supplementary Figure 3D).

### Combination treatment induces upregulation of AXL protein levels in OCI-AML3 cells but not in HL-60 cells

**Figure 6:**
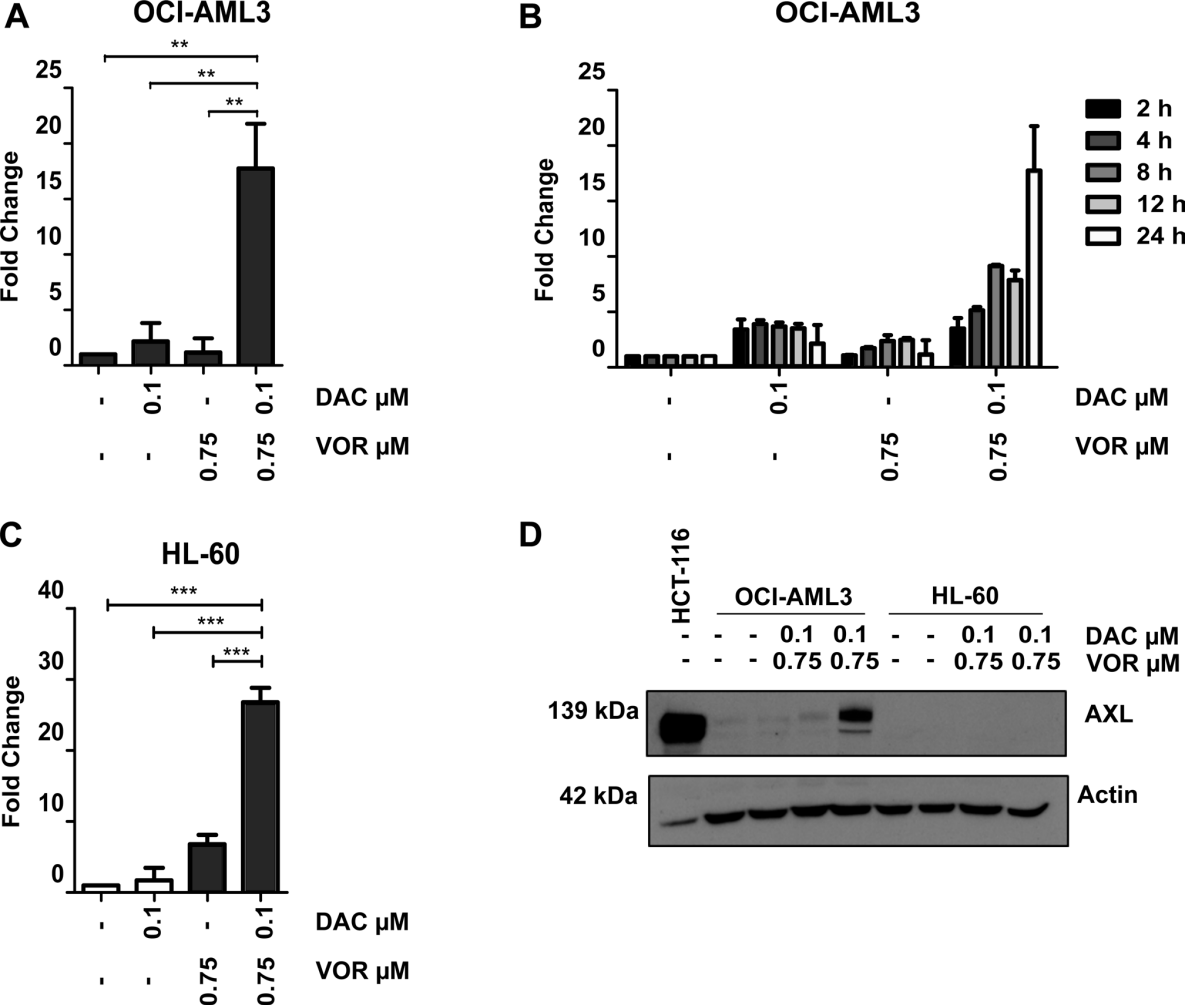
Combined Decitabine and Vorinostat treatment induces AXL expression in AML cell lines OCI-AML3 and HL-60 cells were treated with 0.1 μM DAC, 0.75 μM VOR and the DV combination in a sequential manner. (**A**) qRT-PCR analysis confirmed an increase in *AXL* gene expression in OCI-AML3 cells following DV combined treatment. (**B**) *AXL* expression increased in a time dependent manner (24-hour time course) following the addition of Vorinostat to Decitabine primed OCI-AML3 cells. (**C**) qRT-PCR analysis also confirmed an increase in *AXL* gene expression in the HL-60 cells following DV combined treatment. (**D**) Western blot analysis of total cell lysate confirms an increase in AXL protein levels following DV combination treatment in OCI-AML3 cells. No detectable levels of AXL were found in the HL-60 cells prior to or following treatment. Human colon cancer cell line, HCT-116 cells were used as a positive control for high AXL expression. Actin is used as a loading control. Data represent mean ± SEM; *n* = 3 (***= *p* < 0.001, **= *p* < 0.01).

AXL is known to contribute to cell proliferation, cell survival and drug resistance and was selected for further study. Validation by qRT-PCR confirmed an increase in *AXL* expression (~18 fold) following combination but not by either single agent treatment in OCI-AML3 cells (Figure 6A). Vorinostat treatment of Decitabine primed cells resulted in a continuous increase in expression of *AXL* over the 24-hour time course confirming a combination dependent effect (Figure 6B). This combined treatment increase in *AXL* gene expression was also observed in HL-60 cells (Figure 6C). However, unlike OCI-AML3 cells, the increased gene expression in HL-60 cells did not translate into an increase at the protein level. In fact, AXL protein was undetectable in the HL-60 cell line following all treatments (Figure 6D). The mechanism of AXL turnover in HL-60 cells warrants further investigation.

Not only is *AXL* upregulated in AML cells lines, but the use of an online gene expression tool Bloodspot [24] (Supplementary Figure 4A) has demonstrated that this expression pattern is translated in the clinical setting, where *AXL* is upregulated in a number of AML subgroups compared to normal hematopoietic counterparts.

### H3K9ac contributes to the upregulation of AXL in OCI-AML3 cells

**Figure 7:**
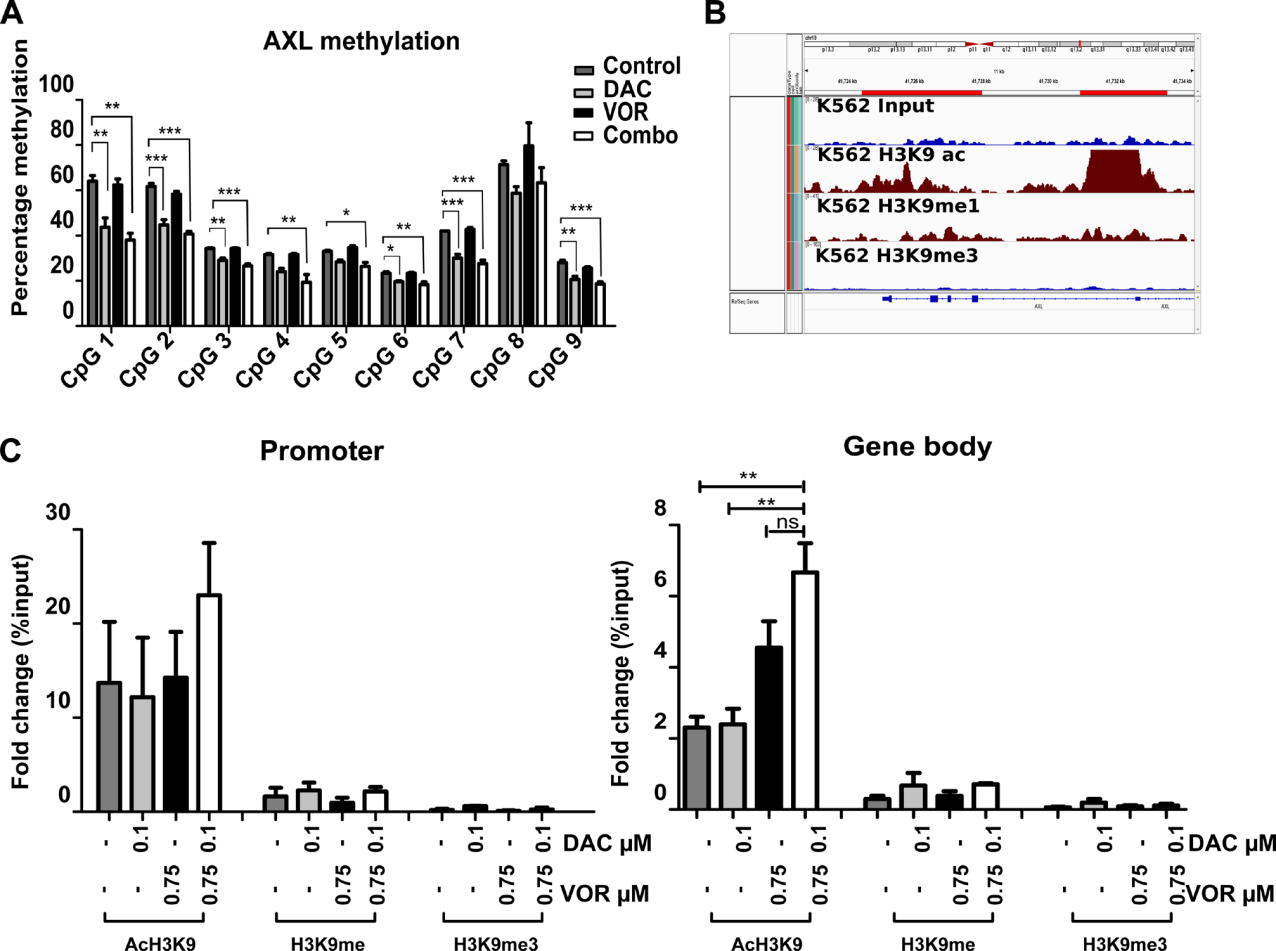
Combined Decitabine and Vorinostat regulate AXL expression through altered DNA and histone epigenetic modifications OCI-AML3 cells were treated with 0.1 μM DAC, 0.75 μM VOR and the DV combination in a sequential manner. (**A**) AXL promoter methylation of bisulfite treated DNA from each treatment group was assessed by pyrosequencing using primers specific to the AXL promoter region. (**B**) Publically available ENCODE data was used to identify the enrichment of epigenetic marks (ac, me and me3) on H3K9 at the AXL promoter and gene body. (**C**) ChIP-qPCR analysis of acH3K9, H3K9me and H3K9me3 localisation at the AXL promoter and gene body in OCI-AML3 cells following treatment. Data represent mean± SEM; *n* = 3 (***= *p* < 0.001; **= *p* < 0.01; *= *p* < 0.05).

Decitabine has previously been reported to alleviate *AXL* promoter methylation and induce re-expression in cell lines [25]. Specific CpG sites, acH3K9 and H3K9me3 marks within the *AXL* gene were further examined to investigate potential mechanisms of regulation following DV treatment in OCI-AML3 cells. As expected, Decitabine alone reduced promoter methylation levels, Vorinostat alone had no significant effect on methylation and the DV combination showed methylation levels similar to Decitabine alone (Figure 7A). This indicates that Vorinostat does not contribute further to the promoter demethylation effects of Decitabine. To further investigate the histone acetylation observed following Vorinostat treatment (Figure 4A and Supplementary Figure 1B) we identified increased acH3K9 activation and decreased H3K9me3 repressive mark status of *AXL* in a myeloid cell background (K562) within the publically available ENCODE [26–28] database (Figure 7B). A series of ChIP-qPCR experiments showed an enrichment of acH3K9 in both the promoter and gene body of AXL following DV combination in OCI-AML3 cells compared to untreated controls. H3K9me3 levels remained low even following DV treatment while levels of H3K9me were moderately enriched following DV combination (Figure 7C).

### BGB324 enhanced the anti-leukemic effect of the Decitabine-Vorinostat combination

To determine if adding the AXL specific inhibitor BGB324 to the DV combination enhanced the therapeutic effect, and as AXL expression was found to increase at 8 hours post Vorinostat addition (Figure 6B); BGB324 was added to the DV treated cells at this time point.

**Figure 8:**
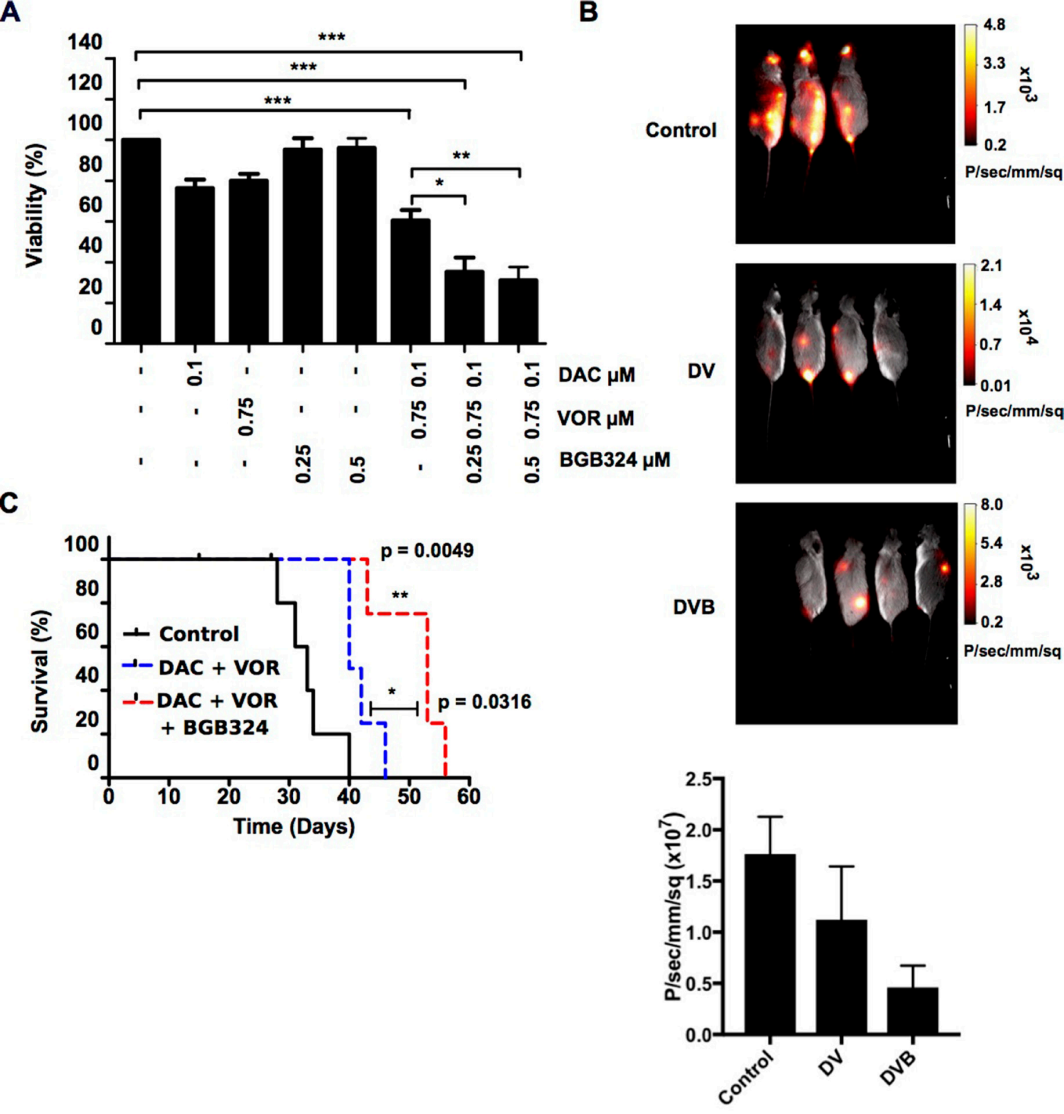
Decitabine/Vorinostat/BGB324 triple combination treatment prolonged the survival and decreased the leukemic burden in mice OCI-AML3 luciferase expressing cells were treated with 0.1 μM DAC, 0.75 μM VOR, 0.5 μM BGB324 and the double DV and triple DVB combination in a sequential manner. (**A**) The viability of OCI-AML3 luciferase cells was assessed using the CellTitre-Glo^®^ assay and represented as percentage of control. Data represent mean ± SEM; *n* = 3 (***= *p* < 0.001; **= *p* < 0.01; *= *p* < 0.05). (**B**) 1.5 × 10^6^ OCI-AML3 luciferase cells (treated *in vitro* with vehicle control, double DV and triple DVB combinations in a sequential manner for 72 hours) were transplanted via tail vein injection into NSG mice (*n* = 5 per treatment group). Bioluminescence imaging commenced 15 days post transplantation. Representative images taken at day 29 post transplantation of surviving mice show the disease burden in mice that received control (top), double DV (middle) and triple DVB (bottom) treated cells. Average luminescence intensity for each cohort is shown as Photons/sec/mm/sq (**C**) Mice were sacrificed at the first sign of illness and Kaplan Meier curves were generated comparing the survival of mice that received control treated OCI-AML3 luciferase cells (black line) those that received cells treated with the double DV (blue dashed line) and triple DVB (red dashed line) combination (**= *p* < 0.01; *= *p* < 0.05).

Low doses of BGB324 alone (0.25 μM and 0.5 μM) had no effect on OCI-AML3 cell viability. However when combined with higher dose DV, 0.25 μM BGB324 and 0.5 μM BGB324 (DVB triple combination) significantly decreased cell viability further than the DV double combination (Figure 8A). As HL-60 cells do not express AXL, BGB324 appears to have a non-specific induced toxicity in HL-60 cells. At the doses used, a reduction in cell viability (~15–20%) was observed. Following DVB treatment there appears to be an additive effect of BGB324 addition rather than a synergistic effect as the viability is reduced by ~20% more than DV treatment alone (Supplementary Figure 5).

To further examine the effects of BGB324 on leukemic cell growth and disease development, luciferase-expressing OCI-AML3 cells were treated *in vitro* with the DVB triple combination and transplanted into NSG recipient mice. Single agent treatment alone had no significant effect on survival of the transplanted mice compared to control (median survival: 33 days) (Supplementary Figure 6). Throughout the time course the double combination treatment arm showed reduced leukemic burden, which was further reduced in the triple DVB combination arm (Figure 8B). Figure 8B also shows a histogram depicting the average luminescence intensity obtained from the control, DV and DVB cohorts following identification of each region of interest-ROI (example of ROI shown in Supplementary Figure 6A). The DV cohort showed reduced luminescence intensity compared to the control; this was further significantly reduced in the DVB cohort. Due to the aggressive nature of the OCI-AML3 cells, leukemia burden was not a determinant of mouse survival. However, mice transplanted with DV treated cells tended to survive longer (n~4 days) than mice receiving vehicle or single agent treatments (Supplementary Figure 6B). A significant extension in survival of mice treated with the triple DVB combination compared to the control group (n~13 days, *p* value = 0.0049) and DV group (n~9 day, *p* value = 0.0316) was observed (Figure 8C). Together, these data support the use of epi-sensitization and multi-step regimens to identify clinically relevant combination therapies to optimize AML patient treatment.

## DISCUSSION

A major advantage of combined epigenetic therapy is the ability to lower the effective drug concentration, with associated reduction in toxicity, whilst increasing the therapeutic efficacy of cancer therapy. This may reduce adverse side effects rendering many current cytotoxic agents more tolerable, in particular for elderly patients, while at the same time improving patient outcome or survival.

A number of studies [7, 9, 29] have assessed the safety, tolerability and anti-leukemic effect of Decitabine and Vorinostat in AML. Reports from clinical trials in AML and other leukemias suggest that patients respond better when treated with a concurrent dosing schedule [9, 29]. In contrast, a phase I clinical study in solid tumors and Non-Hodgkin lymphoma reports that a sequential schedule was more effective [30]. Experiments have also showed greater gene reactivation following sequential treatment with Decitabine followed by the HDAC inhibitor [18, 31]. The principal objective of this study was to assess the combined effects of Decitabine and Vorinostat in AML cells, to investigate the gene expression signature associated with combined treatment and to identify possible epi-sensitized combinations.

Cognizant of the differences in response between clinical scheduling and the *in vitro* studies, we took forward two different regimens; a) Decitabine and Vorinostat administered at day 0 for a total of 72 hours and b) Decitabine administered at day 0 for 48 hours with the addition of Vorinostat at day 2 for 24 hours. Due to the requirement for cell division to incorporate Decitabine, we decided to prime with Decitabine to allow a maintained demethylating effect, followed by Vorinostat treatment to induce acetylation. Since HDAC inhibitor activity is much more transient, we theorized that if performed in reverse (priming with Vorinostat), the increase in acetylation may supersede demethylation too far in advance to have any significant combinatorial effect. Doses used throughout were clinically achievable (as calculated based on Stathis *et al* [30]), and were shown to avoid induction of large scale cytotoxic cell kill, induce DNA global demethylation and histone acetylation as single agent treatments.

CI values indicated the degree of synergistic inhibition of viability between both agents in HL-60 and OCI-AML3 cell lines. Interestingly, cells treated with a sequential dosing regimen tended to have a greater degree of synergy than concurrent treatments. For this reason, we progressed with the sequential regimen to identify potential mechanisms for the observed anti-leukemic effect *in vitro*. In addition, we found the HL-60 cells to have a greater sensitivity to this treatment compared to the OCI-AML3 cells, suggesting that different primary AML subtypes may exhibit differing degrees of sensitivity to such treatments.

With both single agents having previously been shown to induce anti-leukemic effects through mechanisms such as cell cycle arrest, induction of apoptosis and re-expression of silenced genes; we investigated whether the synergistic decrease in viability was due to an enhancement of one or more of these mechanisms. Low doses of Decitabine have little effect on leukemic cell cycle however; higher doses induce a G2/M arrest [32]. Here we support these findings by showing that low micro-molar doses of Decitabine have limited activity on the cell cycle of OCI-AML3 cells while the addition of Vorinostat enhanced the induction of cell death, shown by the increase in Sub G1 population. Although Brodska *et al*. [17] reported similar findings using a concurrent combination of Decitabine and Vorinostat; our study shows that sequential treatment with lower doses can enhance cell kill. Furthermore we show that the increase in Sub G1 population following combined treatment is not merely a cytotoxic affect but that the treatment signals an increase of apoptotic pathways through the cleavage of caspases.

With increasing evidence supporting the interplay between DNA methylation and histone acetylation patterns in regulating gene expression, it is rational to hypothesize that the combined use of Decitabine and Vorinostat will affect the molecular events within the AML cell lines, contribute to alter gene expression signatures as reported by others [18, 31] and activate survival signals to counteract the enhanced anti-leukemic effects. In concordance with others [33, 34], we show that sequential combination of Decitabine and Vorinostat leads to heightened histone acetylation in the OCI-AML3 and HL-60 cell lines. It has been known for some time that methyl binding proteins such as MeCP2 are known to recruit transcriptional repressor complexes which include HDACs, decreasing acetylation and inhibiting gene transcription [35–37]. A reduction in levels of methylation following DNMTi treatment and therefore a reduction in the binding capacity of these HDAC containing repressor complexes provides a potential explanation for the enhanced levels of histone acetylation when these two EMTs are used in combination. Further reductions in DNMT1 protein levels were also found in HL-60 cells, although less pronounced in OCI-AML3 cells, possibly due to the already significantly diminished DNMT1 levels by Decitabine alone. The concept that HDAC inhibitors can decrease DNMT1 expression is not new. A number of publications describe a decrease in DNMT1 at both the mRNA level and protein level with Trichostatin A treatment in cancer models including; hepatocellular, bladder and breast cancer cell lines [38–40]. Zhou *et al*. suggest a possible mechanism whereby DNMT1 is bound to and stabilised by HSP90 and that the addition of Trichostatin A mediates the acetylation of HSP90, disaggregating the complex and destabilizing DNMT1, targeting it for proteasome degradation [40]. They also report that Vorinostat acetylates HSP90 in breast cancer cells providing a possible similar mechanism of action [41]. The reduction of DNMT1 in HL-60 cells did not lead to any further reduction in methylation status as shown by LINE-1 levels; a longer time frame may be needed to assess if the decrease in DNMT1 can lead to a reduction in methylation levels and changes in gene specific methylation which warrants further study.

Gene expression signatures induced following Decitabine, Vorinostat or the DV combined sequential treatments in the OCI-AML3 cell line were determined. As expected, low dose Decitabine (0.1 μM) alone had little effect on gene expression. Decitabine has a short half-life and its effects are reversible, so repeated dosing may be required to maintain hypomethylation and increase efficacy of this agent. Vorinostat alone had a greater effect on gene expression than Decitabine alone. A total of 79 probe-sets had altered expression levels following Vorinostat in comparison to 11 probe-sets being altered with Decitabine treatment. Of these 79 probe-sets, 72 (~91%) were induced compared to 7 (~9%) repressed. Furthermore the DV combination produced a gene expression signature with 140 probe-sets (≡118 genes) significantly upregulated and unique to the combination treatment. This supports the concept that removal of methylation from DNA is essential for the regulation of many genes but alone is insufficient to carry out re-expression and that HDACi's can reactivate genes previously silenced by methylation [18, 31, 42–44] Bioinformatics analysis identified that highly enriched processes were associated with the immune response, the regulation of cell death, regulation of cell proliferation and response to oxidative stress. Amongst the re-expressed genes were *CDKN2A*, *EIF2AK2*, *TNFSF10* and *XAFI*, all of which are associated with cell cycle inhibition or induction of cell death through increasing caspase activity and the reduction of which are known to be associated with the development of leukemia [45–49]. The upregulation of a number of these genes may provide a link to explain some of the enhanced anti-leukemic affects observed throughout this study. interferon response were also upregulated following combined treatment; these included *IFI27*, *IFIH1*, *IFIT2*, *ISG20*, *STAT1*, *STAT2* and *OAS1*, some of which are known Decitabine target genes, albeit at a higher dose [50].

*AXL* was identified as one of the most highly expressed genes following DV treatment (18-fold induction) and was of particular importance as it is implicated in many cancers [51, 52] A number of its oncogenic properties include a role in cell proliferation, survival and inhibition of apoptosis [53, 54]. The use of Bloodspot data indicate that AXL expression is higher in certain subsets of AML compared to normal hematopoietic counterparts while Ben-Batalla *et al*. showed that patients expressing higher than median AXL levels had a worse prognosis [51].

A number of therapeutic agents, including Decitabine, have been reported to upregulate AXL and induce drug resistance [55]. AXL was upregulated with the DV combination treatment in OCI-AML3 and HL-60 cells. Bisulfite pyrosequencing and ChIP-based analysis identified that upregulation of *AXL* was due in part to Decitabine-induced reduction in methylation at the promoter that was permissive for gene-specific acH3K9 activation and reduction of H3K9me3 repression.

The data supports a hypothesis that DV combination treatment of AML cells results in epigenetic induction of AXL expression which may contribute to the partial drug resistance phenotype observed in OCI-AML3 cells, relative to the HL-60 cell line where the same dose DV combination had a greater level of synergistic decrease on cell viability. To test this hypothesis, we challenged the DV-treated OCI-AML3 cells with an AXL specific inhibitor (BGB324) in novel triple combination pre-clinical studies. Triple DVB combination resulted in significantly increased disease-free-survival over vehicle, mono or dual therapies in transplantation models. More recently, BGB324 received orphan drug designation for the use in treating AML [51, 56] and in addition, has shown promise when combined with immune checkpoint inhibitors. AXL and other TAM family members function to dampen the innate immune response through inhibition of toll-like receptors and their signaling cascades [57]. A recent article highlighted a potentially successful therapeutic application of the AXL inhibitor in combination with immune checkpoint inhibitors anti-CTLA-4/anti-PD1 where they stated that these agents induce AXL expression in an attempt to evade immune responses [58]. Our study reports similar findings whereby high AXL expressing DV treated cells increase a number of suppressor of cytokine signalling molecules including SOCS1 and TWIST [57, 59]. Further studies are required to identify if the increase in SOCS1, TWIST and the elevated phospho-ERK1/2 levels following DV treatment are in part attributable to AXL signaling and whether this may be an attempt by the cells to escape eradication through an inflammatory mediated response.

To address whether the mutational status of each cell line could play a role in the sensitivity to BGB324, we performed patient survival analysis on a local data set which incorporated data from the Microarray Innovations in LEukemia (MILE) study [60]. *AXL* expression was correlated with NPM1 and APL status as OCI-AML3 cells harbor mutant NMP1 and HL-60 cells were derived form a patient with APL t(15;17). NPM1 status in conjunction with AXL levels did not appear to have any significant effect on patient survival suggesting that the sensitivity to BGB324 in the OCI-AML3 cell line is likely attributable to the high AXL levels in OCI-AML3 cells and not the mutational characteristics of the cell line. Although it did not reach significance with the number of patients included in our analysis, those with APL and low *AXL* expression tended to have an improved 5-year survival compared to APL patients with high *AXL* expression. This further supports other findings that state high *AXL* expression leads to a worse prognosis in leukemia patients (Supplementary Figure 4B). However, the enhanced sensitivity to DV treatment in the HL-60 cell is more likely explained by the absence of AXL protein/signaling in this cell line. Unlike the cell line, patients harboring this translocation my still retain high *AXL* levels and benefit from this novel triple therapy.

Although currently only patients with high AXL would appear to be candidates for such treatment, we believe that further investigation into combining BGB324 in a triple epigenetic combination therapy with Decitabine and Vorinostat is warranted. Previous studies have shown that Decitabine/Vorinostat combination is safe and well tolerated in patients both alone and followed by re-induction chemotherapy, [7, 9] as is the use of BGB324 as demonstrated through clinical studies in leukemic patients [61]. Phase I clinical studies would be required to assess the safety and tolerability of this triple combination treatment in patients.

In conclusion, this study has characterised the synergistic activity of Decitabine with Vorinostat in *in vitro* AML models and identified a potential biomarker (AXL) and possible mechanisms of action through gene expression profiling. Furthermore, this work has paved the way for further development of a novel triple combination therapy for the treatment of AML.

## MATERIALS AND METHODS

### Materials

Decitabine; 5-aza-2′deoxycitidine (Sigma-Aldrich, Dorset, UK) was dissolved in water. Vorinostat; SAHA (SelleckChem, Houston, USA) was dissolved in DMSO. BGB324 was a gift from BerGenBio (Bergen, Norway) and was dissolved in DMSO; final DMSO for *in vitro* assays was 0.1%.

### Cell lines

OCI-AML3 cells (Deutsche Sammlung von Mikroorganismen und Zellkulturen GmbH, Braunschweig, Germany), and HL-60 cells (American Type Culture Collection, Middlesex, UK) were maintained in RPMI-1640 media supplemented with 10% fetal calf serum, 1% Pen/Strep and incubated in 5% CO_2_ at 37^°^C.

### Dosing schedule for combination treatment

Concurrent treatment consisted of Decitabine and Vorinostat added at day 0 for 72 hours while sequential treatment consisted of Decitabine added at day 0 for 48 hours and Vorinostat added at 48 hours for a further 24 hours. Triple combination treatment: cells were treated as above for the sequential combination of Decitabine and Vorinostat with BGB324 added 8 hours following Vorinostat. Single agent controls were treated for the time course stated above. Cells were harvested at 72 hours for processing and downstream analysis. Only sequential Decitabine and Vorinostat combination dosing is referred to as DV throughout.

### Viability, caspase activity assays and combination index calculation

Cell viability and caspase activity was determined using the CellTitre-Glo^®^ luminescent viability kit and caspase Glo^®^ assays (Promega, Madison, USA) as described in the manufacturer's instructions. Calcusyn Software Version 2.1 (Cambridge Computing Imaging Ltd, Cambridge, UK) was used to calculate the combination index (CI) using the percentage of cells affected. The Chou-Talalay method [62] is employed by Calcusyn for calculating the CI. The software uses a method based on the median effect equation (MEE) derived from the mass action law and correlates dose and effect to produce the CI value; anything less than one showing a level of synergy (closer the number to 0 shows a greater degree of synergy) or a number greater than 1 showing antagonism between the agents. In this case we used the percentage of non-viable cells after treatment (as determined by the CellTitre-Glo^®^ assay described above) as the fractional affect. This data is input into the software along with the dose of all agents used in the combination treatment.

### FACS analysis

To assess cell cycle changes, cells were harvested and re-suspended in 70% ice-cold ethanol overnight followed by staining with propidium iodide mix (40 μg/mL PI, 2 ng/ml RNase A). BD FITC Annexin V apoptosis detection kit (BD Biosciences, California, USA) was used according to the manufacturer's instructions to assess apoptosis. Samples were analysed using LSRII and BD FACSDiva software.

### Western blot analysis

Cells were washed and lysed using RIPA buffer (50 mM Tris (pH 7.4), 150 mM NaCl, 5 mM EDTA, 1% triton X(X100) and 0.1 % SDS) containing protease inhibitor cocktail tablets (Roche Products Ltd, Welwyn Garden City, UK) and phosphatase inhibitors (sodium fluoride and sodium orthovanadate). Protein concentration was quantified according to the manufacturer's instructions (Pierce BCA Protein Assay kit, Thermo Fisher Scientific, USA). Total lysates were prepared by adding 10X loading buffer to 30 μg of total lysate before denaturation for 5 minutes at 95°C. Lysates were then electrophoresed on various percentage Tris Glycine gels and immunoblotted using anti-caspase 8 (Enzo Life Sciences, UK), anti-caspase 9, anti-caspase 3, anti-acetylated tubulin, anti-DNMT1, anti-AXL (Cell signalling, Massachusetts, USA), anti-hyperacetylated histone H4 (Merck Millipore, UK) and anti-PARP (eBioscience, UK) antibodies.

### Bisulfite pyrosequencing for global DNA methylation analysis

DNA was extracted and bisulfite treated using the DNeasy Blood and Tissue Kit and the Epitect Bisulfite Kit (Qiagen, Crawley, UK) according to the manufacturer's instructions. Bisulfite DNA underwent PCR using primers for the human LINE-1 sequence (Qiagen) and samples were sequenced using the Pyromark Q24 Pyrosequencer.

### Analysis of gene expression by microarray

RNA was extracted from biological triplicate samples using the RNeasy kit. A maximum of 8 μg of total RNA was processed to cRNA and hybridised to Affymetrix Human Genome U133 Plus 2.0 Arrays following the protocol outlined by Roche Diagnostics (Burgess Hill, UK). Arrays were then stained using an Affymetrix GeneChip Fluidics Station 450 and scanned using the Affymetrix GeneChip Scanner 3000 5G. Analysis of data was performed using Partek Genomic Suite Software (Partex Incorporated, Missouri, USA). Following CEL file importation to Partek Genomic Suite, data were analysed using a Robust Multi-array (RMA) analysis to correct for background, quantile normalise and summarise the probe set intensity into expression measurements. Data was subjected to ANOVA analysis and subsequent gene lists were created based on the following criteria.

- Vehicle control v Decitabine (FDR *p* < 0.05 and fold change or ≤ –2 or ≥ 2)
- Vehicle control v Vorinostat (FDR *p* < 0.01 and fold change ≤ –4 or ≥ 4)
- Vehicle control v Combination (FDR *p* < 0.01 and fold change ≤ –4 or ≥ 4)

### Quantitative real time PCR

RNA was extracted using the RNeasy Kit (Qiagen) as described by the manufacturer's protocols and reverse transcribed using the High-Capacity cDNA reverse transcription kit (Applied Biosystems, Life Technologies, UK).

SYBR Green real time quantitative PCR was performed using the 7900HT Real-time PCR system under optimal cycling conditions. Primers used for mRNA quantification were designed in-house using Primer-Blast and purchased from Eurofins Genomic (Eurofins Genomic, Ebersberg, Germany). Primer sequences used can be found in Supplementary Table 3. Validation of microarray studies was carried out using RNA matched samples to those used for hybridisation to the Affymetrix arrays.

Gene expression is shown as fold change of the control treatment and normalised to the endogenous controls. The Ct value for each gene was normalised to β-Actin and 18S endogenous controls (ΔCt), ΔCt was then normalised to the treatment control (ΔΔCt), and the ΔΔCt was used to calculate gene expression changes as a fold change over control.

### ChIP-qPCR

Combination treatments were set up as described in the methods section. 1 × 10^6^ cells per IP were harvested and fixed in 1% formaldehyde. Cells were washed and chromatin was isolated; fixed samples were resuspended in LB1 buffer (50 mM Hepes-KOH, pH 7.5, 140 mM NaCl, 1 mM EDTA, 10% glycerol, 0.5% IGEPAL, 0.25% Triton X-100) for 10 minutes, pelleted and resuspended in LB2 buffer (10 mM TrisHCl, pH8, 200 mM NaCl, 1 mM EDTA, 0.5 mM EGTA) for 5 minutes, pelleted and resuspended in LB3 buffer (10 mM TrisHCl, pH8, 100 mM NaCl, 1 mM EDTA, 0.5 mM EGTA, 0.1% NA-deoxycholate, 0.5% N-laurylsarcosine). Cells were sonicated for appropriate time to obtain fragments of between 200-500 bp. Following sonication, 10% triton X-100 was added and chromatin was snap frozen for chromatin immunoprecipitation studies. 30 μL beads per IP were washed and blocked overnight at 4°C in sterile 0.5% BSA:PBS. Chromatin was immunoprecipitated with chosen antibodies (anti-histone H3 (acetyl K9), anti-histone H3 (mono methyl K9) and anti-histone H3 (tri methyl K9) (Abcam, UK)) and eluted using elution buffer (50 mM Tris pH8, 10 mM EDTA, 1% SDS). Chromatin was purified using the QIAquick PCR purification kit (Qiagen) as described in the manufacturer's instructions.

### *Ex-vivo* studies

Animal handling was in line with the guidelines set out by the UK Animals Scientific Procedures Act 1986. The Ethical Review Committee for animal research at Queen's University Belfast approved all experimental procedures. The OCI-AML3 luciferase cell line was created as follows. OCI-AML3 cells were seeded the day before transfection at 5 × 10^6^ cells/10 cm dish and grown to approximately 70–80% confluency. Transfection of 293T cells was performed using Turbofect (Thermo Scientific, Massachusetts, USA) with a molar of 1:3:6 ratio of VSV-G envelope vector pMD2.G, packaging vector psPAX2 (kindly provided by Dr Fabio Liberante) and firefly luciferase vector pSLIEW (kindly provided by Prof Olaf Heidenreich). Viral supernatant was harvested and filtered 72 hours after transfection. A volume of 500 μl filtered pSLIEW virus was added to 2 × 10^5^ cells in 200 μl medium with 8 μg/ml Polybrene (Sigma Aldrich) and incubated for 48 hours before selection in neomycin. Validation of OCI-AML3 transduction was performed using Bruker *In Vivo* Xtreme imaging system before cells were treated and transplanted. Cells were treated *in vitro* with control, each single agent and either 0.1 μM Decitabine plus 0.75 μM Vorinostat or 0.1 μM Decitabine plus 0.75 μM Vorinostat plus 0.5 μM BGB324. A total of 1.5 × 10^6^ cells per mouse were resuspended in PBS and transplanted via tail vein injection into recipient NSG mice (Charles River, Tranent, UK) - (each treatment group *n* = 5). Mice were imaged for luciferase signal using the Bruker *In Vivo* Xtreme imaging system every second day between day 15 and day 37. Mice were sacrificed at the first sign of disease and survival extension was assessed.

### Statistical analysis

All statistical analysis was performed using Graphpad Prism version 5.00 for Windows (California, USA). All statistical tests were performed on at least a biological replicate of 3.

## SUPPLEMENTARY MATERIALS FIGURES AND TABLES



## References

[1] Burnett A, Wetzler M, Löwenberg B (2011). Therapeutic advances in acute myeloid leukemia. J Clin Oncol.

[2] Yates JW, Wallace HJ, Ellison RR, Holland JF (1973). Cytosine arabinoside (NSC-63878) and daunorubicin (NSC-83142) therapy in acute nonlymphocytic leukemia. Cancer Chemother Rep.

[3] Fong CY, Morison J, Dawson MA (2014). Epigenetics in the hematologic malignancies. Haematologica.

[4] Cameron EE, Bachman KE, Myöhänen S, Herman JG, Baylin SB (1999). Synergy of demethylation and histone deacetylase inhibition in the re-expression of genes silenced in cancer. Nat Genet.

[5] FDA Approval for Decitabine (2013). http://www.cancer.gov/cancertopics/druginfo/fda-decitabine.

[6] Saba H (2009). Decitabine in myeloid malignancies. Hematology Meeting Reports.

[7] Burke MJ, Lamba JK, Pounds S, Cao X, Ghodke-Puranik Y, Lindgren BR, Weigel BJ, Verneris MR, Miller JS (2014). A therapeutic trial of decitabine and vorinostat in combination with chemotherapy for relapsed/refractory acute lymphoblastic leukemia. Am J Hematol.

[8] Issa JP, Garcia-Manero G, Huang X, Cortes J, Ravandi F, Jabbour E, Borthakur G, Brandt M, Pierce S, Kantarjian HM (2015). Results of phase 2 randomized study of low-dose decitabine with or without valproic acid in patients with myelodysplastic syndrome and acute myelogenous leukemia. Cancer.

[9] Kirschbaum M, Gojo I, Goldberg SL, Bredeson C, Kujawski LA, Yang A, Marks P, Frankel P, Sun X, Tosolini A, Eid JE, Lubiniecki GM, Issa JP (2014). A phase 1 clinical trial of vorinostat in combination with decitabine in patients with acute myeloid leukaemia or myelodysplastic syndrome. Br J Haematol.

[10] Plumb JA, Strathdee G, Sludden J, Kaye SB, Brown R (2000). Reversal of drug resistance in human tumor xenografts by 2′-deoxy-5-azacytidine-induced demethylation of the hMLH1 gene promoter. Cancer Res.

[11] Juergens RA, Wrangle J, Vendetti FP, Murphy SC, Zhao M, Coleman B, Sebree R, Rodgers K, Hooker CM, Franco N, Lee B, Tsai S, Delgado IE (2011). Combination epigenetic therapy has efficacy in patients with refractory advanced non-small cell lung cancer. Cancer Discov.

[12] Bhatla T, Wang J, Morrison DJ, Raetz EA, Burke MJ, Brown P, Carroll WL (2012). Epigenetic reprogramming reverses the relapse-specific gene expression signature and restores chemosensitivity in childhood B-lymphoblastic leukemia. Blood.

[13] Falkenberg KJ, Gould CM, Johnstone RW, Simpson KJ, Duvic M, Olsen EA, Fantin VR, Lindemann RK, Fotheringham S, Khan O, Nijwening JH, Beijersbergen RL, Berns K (2014). Genome-wide functional genomic and transcriptomic analyses for genes regulating sensitivity to vorinostat. Sci Data.

[14] Dupéré-Richer D, Kinal M, Pettersson F, Miller WH (2013). >Resistance To Vorinostat In Hematological Malignancies May Involve Cytoprotective UPR and Correlates With Increased Sensitivity To Bortezomib-Induced Cell Death. Blood.

[15] Fenaux P, Mufti GJ, Hellström-Lindberg E, Santini V, Gattermann N, Germing U, Sanz G, List AF, Gore S, Seymour JF, Dombret H, Backstrom J, Zimmerman L (2010). Azacitidine prolongs overall survival compared with conventional care regimens in elderly patients with low bone marrow blast count acute myeloid leukemia. J Clin Oncol.

[16] Sadashiv SK, Hilton C, Khan C, Rossetti JM, Benjamin HL, Fazal S, Sahovic E, Shadduck RK, Lister J (2014). Efficacy and tolerability of treatment with azacitidine for 5 days in elderly patients with acute myeloid leukemia. Cancer Med.

[17] Brodská B, Holoubek A, Otevřelová P, Kuželová K (2013). Combined treatment with low concentrations of decitabine and saha causes cell death in leukemic cell lines but not in normal peripheral blood lymphocytes. Biomed Res Int.

[18] Kalac M, Scotto L, Marchi E, Amengual J, Seshan VE, Bhagat G, Ulahannan N, Leshchenko VV, Temkin AM, Parekh S, Tycko B, O’Connor OA (2011). HDAC inhibitors and decitabine are highly synergistic and associated with unique gene-expression and epigenetic profiles in models of DLBCL. Blood.

[19] Chen MY, Liao WS, Lu Z, Bornmann WG, Hennessey V, Washington MN, Rosner GL, Yu Y, Ahmed AA, Bast RC (2011). Decitabine and suberoylanilide hydroxamic acid (SAHA) inhibit growth of ovarian cancer cell lines and xenografts while inducing expression of imprinted tumor suppressor genes, apoptosis, G2/M arrest, and autophagy. Cancer.

[20] Brodská B, Otevřelová P, Holoubek A (2011). Decitabine-induced apoptosis is derived by Puma and Noxa induction in chronic myeloid leukemia cell line as well as in PBL and is potentiated by SAHA. Mol Cell Biochem.

[21] Yang D, Torres CM, Bardhan K, Zimmerman M, McGaha TL, Liu K (2012). Decitabine and vorinostat cooperate to sensitize colon carcinoma cells to Fas ligand-induced apoptosis *in vitro* and tumor suppression *in vivo*. J Immunol.

[22] Huang W, Sherman BT, Lempicki RA (2009). Systematic and integrative analysis of large gene lists using DAVID bioinformatics resources. Nat Protoc.

[23] Huang W, Sherman BT, Lempicki RA (2009). Bioinformatics enrichment tools: paths toward the comprehensive functional analysis of large gene lists. Nucleic Acids Res.

[24] Bagger FO, Sasivarevic D, Sohi SH, Laursen LG, Pundhir S, Sønderby CK, Winther O, Rapin N, Porse BT (2016). BloodSpot: a database of gene expression profiles and transcriptional programs for healthy and malignant haematopoiesis. Nucleic Acids Res.

[25] Mudduluru G, Allgayer H (2008). The human receptor tyrosine kinase Axl gene—promoter characterization and regulation of constitutive expression by Sp1, Sp3 and CpG methylation. Biosci Rep.

[26] ENCODE Project Consortium (2012). An integrated encyclopedia of DNA elements in the human genome. Nature.

[27] Thorvaldsdóttir H, Robinson JT, Mesirov JP (2013). Integrative Genomics Viewer (IGV): high-performance genomics data visualization and exploration. Brief Bioinform.

[28] Robinson JT, Thorvaldsdóttir H, Winckler W, Guttman M, Lander ES, Getz G, Mesirov JP (2011). Integrative genomics viewer. Nat Biotechnol.

[29] How J, Minden MD, Brian L, Chen EX, Brandwein J, Schuh AC, Schimmer AD, Gupta V, Webster S, Degelder T, Haines P, Stayner LA, McGill S (2015). A phase I trial of two sequence-specific schedules of decitabine and vorinostat in patients with acute myeloid leukemia. Leuk Lymphoma.

[30] Stathis A, Hotte SJ, Chen EX, Hirte HW, Oza AM, Moretto P, Webster S, Laughlin A, Stayner LA, McGill S, Wang L, Zhang WJ, Espinoza-Delgado I (2011). Phase I study of decitabine in combination with vorinostat in patients with advanced solid tumors and non-Hodgkin's lymphomas. Clin Cancer Res.

[31] Momparler RL, Côté S, Momparler LF, Idaghdour Y (2014). Epigenetic therapy of acute myeloid leukemia using 5-aza-2′-deoxycytidine (decitabine) in combination with inhibitors of histone methylation and deacetylation. Clin Epigenetics.

[32] Hollenbach PW, Nguyen AN, Brady H, Williams M, Ning Y, Richard N, Krushel L, Aukerman SL, Heise C, MacBeth KJ (2010). A Comparison of Azacitidine and Decitabine Activities in Acute Myeloid Leukemia Cell Lines. PLoS One.

[33] Yang H, Hoshino K, Sanchez-Gonzalez B, Kantarjian H, Garcia-Manero G (2005). Antileukemia activity of the combination of 5-aza-2′-deoxycytidine with valproic acid. Leuk Res.

[34] Zhu WG, Lakshmanan RR, Beal MD, Otterson GA (2001). DNA methyltransferase inhibition enhances apoptosis induced by histone deacetylase inhibitors. Cancer Res.

[35] Nan X, Campoy FJ, Bird A (1997). MeCP2 is a transcriptional repressor with abundant binding sites in genomic chromatin. Cell.

[36] Nan X, Ng HH, Johnson CA, Laherty CD, Turner BM, Eisenman RN, Bird A (1998). Transcriptional repression by the methyl-CpG-binding protein MeCP2 involves a histone deacetylase complex. Nature.

[37] Jones PL, Veenstra GJ, Wade PA, Vermaak D, Kass SU, Landsberger N, Strouboulis J, Wolffe AP (1998). Methylated DNA and MeCP2 recruit histone deacetylase to repress transcription. Nat Genet.

[38] Arzenani MK, Zade AE, Ming Y, Vijverberg SJ, Zhang Z, Khan Z, Sadique S, Kallenbach L, Hu L, Vukojević V, Ekström TJ (2011). Genomic DNA hypomethylation by histone deacetylase inhibition implicates DNMT1 nuclear dynamics. Mol Cell Biol.

[39] Ou JN, Torrisani J, Unterberger A, Provençal N, Shikimi K, Karimi M, Ekström TJ, Szyf M (2007). Histone deacetylase inhibitor Trichostatin A induces global and gene-specific DNA demethylation in human cancer cell lines. Biochem Pharmacol.

[40] Zhou Q, Agoston AT, Atadja P, Nelson WG, Davidson NE (2008). Inhibition of histone deacetylases promotes ubiquitin-dependent proteasomal degradation of DNA methyltransferase 1 in human breast cancer cells. Mol Cancer Res.

[41] Zhou Q, Chaerkady R, Shaw PG, Kensler TW, Pandey A, Davidson NE (2010). Screening for therapeutic targets of vorinostat by SILAC-based proteomic analysis in human breast cancer cells. Proteomics.

[42] Si J, Boumber YA, Shu J, Qin T, Ahmed S, He R, Jelinek J, Issa JP (2010). Chromatin remodeling is required for gene reactivation after decitabine-mediated DNA hypomethylation. Cancer Res.

[43] Paul TA, Bies J, Small D, Wolff L (2010). Signatures of polycomb repression and reduced H3K4 trimethylation are associated with p15INK4b DNA methylation in AML. Blood.

[44] Flotho C, Claus R, Batz C, Schneider M, Sandrock I, Ihde S, Plass C, Niemeyer CM, Lübbert M (2009). The DNA methyltransferase inhibitors azacitidine, decitabine and zebularine exert differential effects on cancer gene expression in acute myeloid leukemia cells. Leukemia.

[45] Soncini M, Santoro F, Gutierrez A, Frigè G, Romanenghi M, Botrugno OA, Pallavicini I, Pelicci P, Di Croce L, Minucci S (2013). The DNA demethylating agent decitabine activates the TRAIL pathway and induces apoptosis in acute myeloid leukemia. Biochim Biophys Acta.

[46] Zhu LM, Shi DM, Dai Q, Cheng XJ, Yao WY, Sun PH, Ding Y, Qiao MM, Wu YL, Jiang SH, Tu SP (2014). Tumor suppressor XAF1 induces apoptosis, inhibits angiogenesis and inhibits tumor growth in hepatocellular carcinoma. Oncotarget.

[47] Guo SX, Taki T, Ohnishi H, Piao HY, Tabuchi K, Bessho F, Hanada R, Yanagisawa M, Hayashi Y (2000). Hypermethylation of p16 and p15 genes and RB protein expression in acute leukemia. Leuk Res.

[48] Merlo A, Herman JG, Mao L, Lee DJ, Gabrielson E, Burger PC, Baylin SB, Sidransky D (#x2032). CpG island methylation is associated with transcriptional silencing of the tumour suppressor p16/CDKN2/MTS1 in human cancers. Nat Med.

[49] Falschlehner C, Emmerich CH, Gerlach B, Walczak H (2007). TRAIL signalling: decisions between life and death. Int J Biochem Cell Biol.

[50] Moreaux J, Rème T, Leonard W, Veyrune JL, Requirand G, Goldschmidt H, Hose D, Klein B (2012). Development of gene expression-based score to predict sensitivity of multiple myeloma cells to DNA methylation inhibitors. Mol Cancer Ther.

[51] Ben-Batalla I, Schultze A, Wroblewski M, Erdmann R, Heuser M, Waizenegger JS, Riecken K, Binder M, Schewe D, Sawall S, Witzke V, Cubas-Cordova M, Janning M (2013). Axl, a prognostic and therapeutic target in acute myeloid leukemia mediates paracrine crosstalk of leukemia cells with bone marrow stroma. Blood.

[52] Wu X, Liu X, Koul S, Lee CY, Zhang Z, Halmos B (2014). AXL kinase as a novel target for cancer therapy. Oncotarget.

[53] Shiozawa Y, Pedersen EA, Patel LR, Ziegler AM, Havens AM, Jung Y, Wang J, Zalucha S, Loberg RD, Pienta KJ, Taichman RS (2010). GAS6/AXL axis regulates prostate cancer invasion, proliferation, and survival in the bone marrow niche. Neoplasia.

[54] Lee-Sherick AB, Eisenman KM, Sather S, McGranahan A, Armistead PM, McGary CS, Hunsucker SA, Schlegel J, Martinson H, Cannon C, Keating AK, Earp HS, Liang X (2013). Aberrant Mer receptor tyrosine kinase expression contributes to leukemogenesis in acute myeloid leukemia. Oncogene.

[55] Wang C, Jin H, Wang N, Fan S, Wang Y, Zhang Y, Wei L, Tao X, Gu D, Zhao F, Fang J, Yao M, Qin W (2016). Gas6/Axl Axis Contributes to Chemoresistance and Metastasis in Breast Cancer through Akt/GSK-3β/β-catenin Signaling. Theranostics.

[56] Sheridan C (2013). First Axl inhibitor enters clinical trials. Nat Biotechnol.

[57] Rothlin CV, Ghosh S, Zuniga EI, Oldstone MB, Lemke G (2007). TAM receptors are pleiotropic inhibitors of the innate immune response. Cell.

[58] Gausdal G, Davidsen K, Wnuk-Lipinska K, Wiertel K, Hellesøy M, Blø M, Ahmed L, Hodneland L, Kiprijanov S, Brekken RA, Lorens JB (2016). Abstract B014: BGB324, a selective small molecule inhibitor of the receptor tyrosine kinase AXL, enhances immune checkpoint inhibitor efficacy. Cancer Immunol Res.

[59] Sharif MN, Sosic D, Rothlin CV, Kelly E, Lemke G, Olson EN, Ivashkiv LB (2006). Twist mediates suppression of inflammation by type I IFNs and Axl. J Exp Med.

[60] Kohlmann A, Kipps TJ, Rassenti LZ, Downing JR, Shurtleff SA, Mills KI, Gilkes AF, Hofmann WK, Basso G, Dell'orto MC, Foà R, Chiaretti S, De Vos J (2008). An international standardization programme towards the application of gene expression profiling in routine leukaemia diagnostics: the Microarray Innovations in LEukemia study prephase. Br J Haematol.

[61] Loges S, Gjertsen BT, Heuser M, Ben-Batalla I, Micklem D, Jorg C, Kebenko M, Fiedler WM, Cortes JE (2016). A first-in-patient phase I study of BGB324, a selective Axl kinase inhibitor in patients with refractory/relapsed AML and high-risk MDS. J Clin Oncol.

[62] Chou TC (2010). Drug combination studies and their synergy quantification using the Chou-Talalay method. Cancer Res.

