# Supplementary material for: Decitabine-Vorinostat combination treatment in acute myeloid leukemia activates pathways with potential for novel triple therapy
Source: Oncotarget. 2017 May 19;8(31):51429–46. doi: 10.18632/oncotarget.18009 (PMC5584259; doi:10.18632/oncotarget.18009)
Supplement: Supplementary file 1 [file oncotarget-08-51429-s001.pdf]

# Decitabine-Vorinostat combination treatment in acute myeloid leukemia activates pathways with potential for novel triple therapy

## SUPPLEMENTARY MATERIALS

**Supplementary Table 1: Combination index for HL-60 and OCI-AML3 cells treated with sequential DV combination**

| | | | HL-60 | | | OCI-AML3 | |
|---|---|---|---|---|---|---|---|
| **DAC** | **VOR** | **CI 1** | **CI 2** | **CI 3** | **CI 1** | **CI 2** | **CI 3** |
| **0.1** | **1** | 0.23 | 0.37 | 0.2 | 0.08 | 1 | 0.24 |
| | **0.75** | 0.21 | 0.54 | 0.21 | 0.23 | 0.9 | 0.43 |
| | **0.5** | 0.36 | 0.55 | 0.2 | 0.44 | 0.93 | 0.4 |
| | **0.25** | 0.47 | 0.89 | 0.5 | 1.19 | 0.9 | 0.3 |
| **0.2** | **1** | 0.14 | 0.54 | 0.15 | 0.04 | 0.91 | 0.3 |
| | **0.75** | 0.22 | 0.55 | 0.1 | 0.12 | 0.65 | 0.46 |
| | **0.5** | 0.39 | 0.62 | 0.11 | 0.2 | 0.84 | 0.49 |
| | **0.25** | 0.62 | 0.57 | 0.08 | 0.37 | 0.51 | 0.58 |
| **0.4** | **1** | 0.15 | 0.54 | 0.14 | 0.03 | 0.52 | 0.36 |
| | **0.75** | 0.22 | 0.59 | 0.12 | 0.08 | 0.6 | 0.5 |
| | **0.5** | 0.53 | 0.64 | 0.12 | 0.26 | 0.5 | 0.7 |
| | **0.25** | 0.6 | 0.6 | 0.09 | 0.51 | 0.41 | 0.91 |

*CI 1-3 = biological triplicates.*

**Supplementary Table 2: Number of probe-sets/genes significantly differentially expressed following each treatment in OCI-AML3 cells**

| | Treatment upregulated relative to the control | |
|---|---|---|
| | Number of probe-sets | Number of genes |
| Decitabine | 11 | 10 |
| Vorinostat | 72 | 63 |
| Combination | 201 | 163 |
| Total | 284 | 236 |
| | Treatment downregulated relative to the control | |
| | Number of probe-sets | Number of genes |
| Decitabine | 0 | 0 |
| Vorinostat | 7 | 6 |
| Combination | 32 | 29 |
| Total | 39 | 35 |

**Supplementary Table 3: Primer sequences**

| | Primer Sequences |
|---|---|
| mRNA | |
| AXL_Forward | GACTCTGGGAGAGGGAGAGTTT |
| AXL_Reverse | CATGCAGACCGCTTCACTCA |
| Genomic | |
| AXL_Promoter_Forward | CCAGGCAGGCAGTGCCAAAT |
| AXL_Promoter_Reverse | TGCCAAACTTTCCTCAGAAGTTGT |
| AXL_Gene Body_Forward | CGCGTAAACAACACGCAGAA |
| AXL_Gene Body_Reverse | TGGGACCAAGTCTTGTAAGTGC |

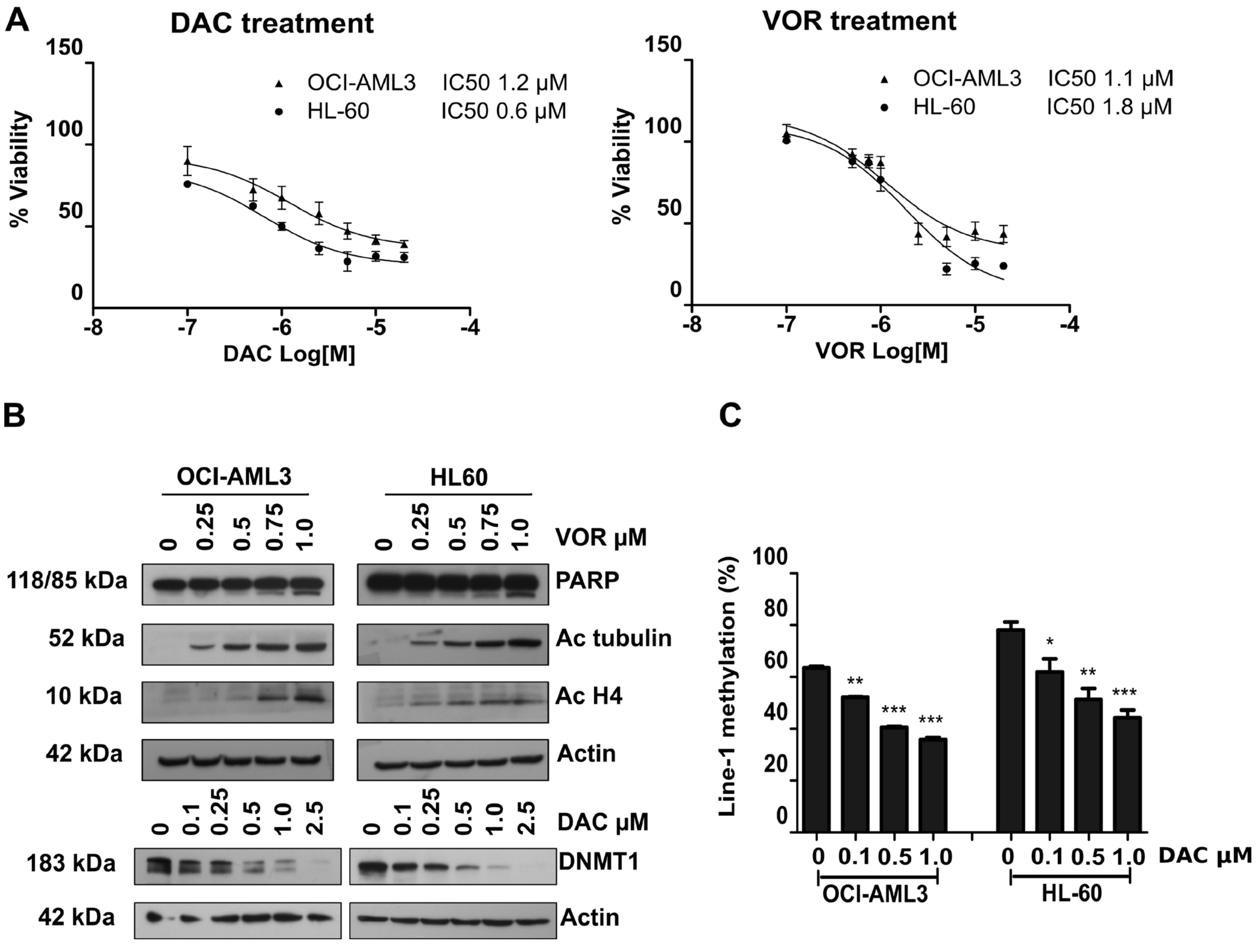


**Supplementary Figure 1: Single agent Decitabine or Vorinostat treatment induces anti-leukemic effects on AML cell lines.** OCI-AML3 and HL-60 cells were treated with increasing doses of DAC or VOR. (**A**) The half maximal inhibitory concentration (IC50) curves for OCI-AML3 and HL-60 cells treated for 72 hour with DAC (left) or 24 hour with VOR (right) as measured by CellTitre-Glo® assay. IC50 values are recorded on each graph. (**B**) Western blot analysis of PARP cleavage, acetylated tubulin and acetylated H4 levels following 24 hour VOR treatment in OCI-AML3 (left) and HL-60 (right) cells. Western blot analysis of DNMT1 levels following DAC treatment. (**C**) Bisulfite converted DNA was analysed and CpG methylation of the human LINE-1 sequence was quantified by pyrosequencing to estimate levels of global DNA methylation following increasing DAC treatment in OCI-AML3 and HL-60 cells. Data represent mean ± SEM; $n = 3$ (***= $p < 0.001$; **= $p < 0.01$; *= $p < 0.05$).

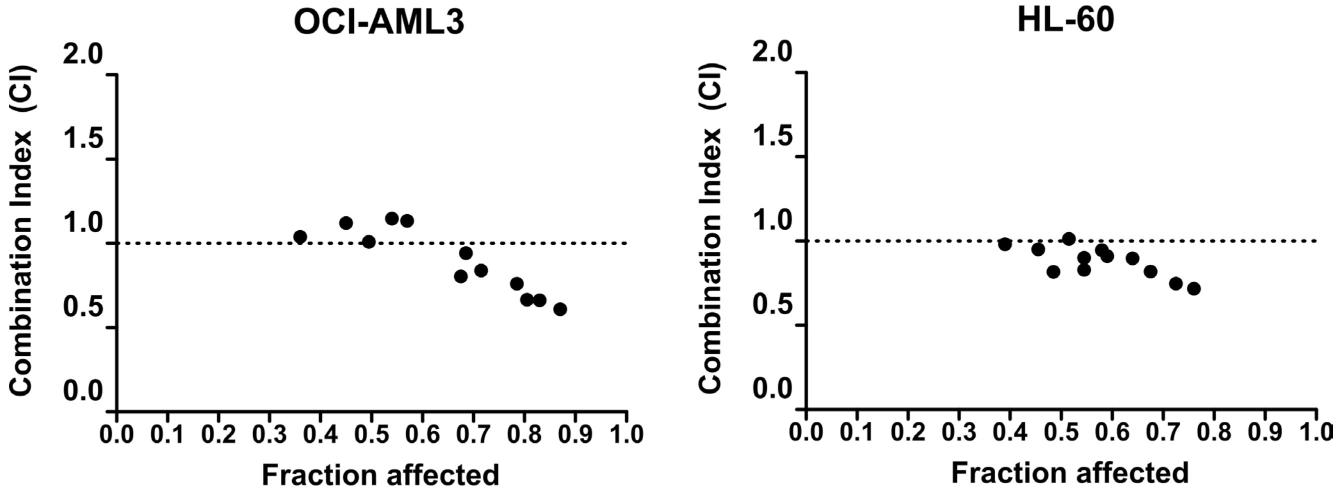


**Supplementary Figure 2: Concurrent Decitabine and Vorinostat treatment induced a low degree of synergy in AML cell lines.** OCI-AML3 (left) and HL-60 (right) cells were treated with DAC (0.1 µM, 0.2 µM and 0.4 µM), VOR (0.25 µM, 0.5 µM, 0.75 µM and 1 µM) and all DV combination doses in a concurrent manner for 72 hours. Cell viability was measured using a CellTitre-Glo® assay and viability percentage was used to calculate the combination index by Calcusyn software. Data represent mean ± SEM; *n* = 3.

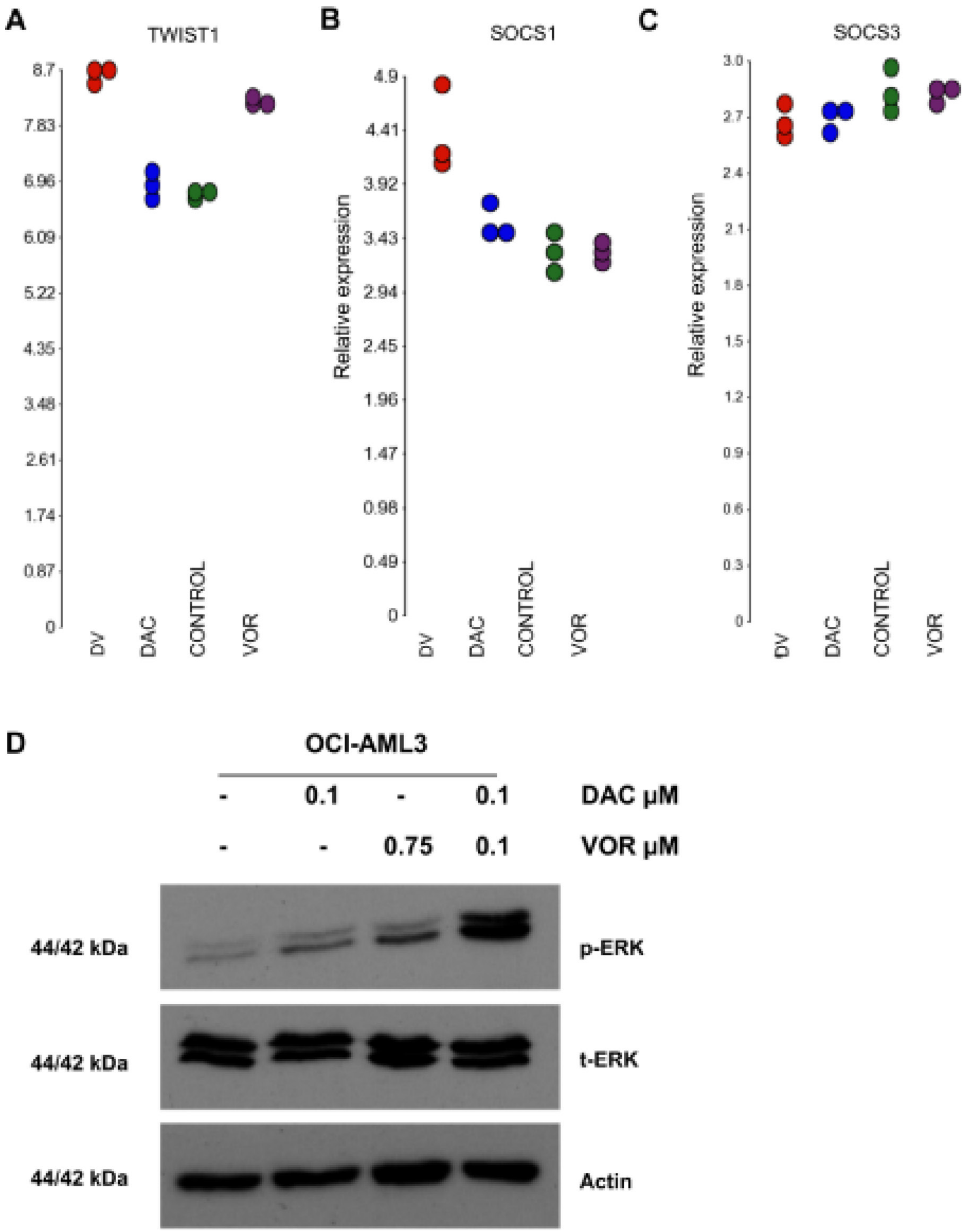


**Supplementary Figure 3: OCI-AML3 cells treated with Decitabine and Vorinostat increase AXL associated genes.** qRT-PCR validation obtained from the microarray analysis of OCI-AML3 cells treated with control, DAC (0.1 µM), VOR (0.75 µM) or DV combination. DV treatment showed an increase in the relative expression of (**A**) TWIST and (**B**) SOCS1 but not in (**C**) SOCS3 in comparison to the control treated cells. (**D**) DV combination treatment increase levels of phospho-ERK1/2.

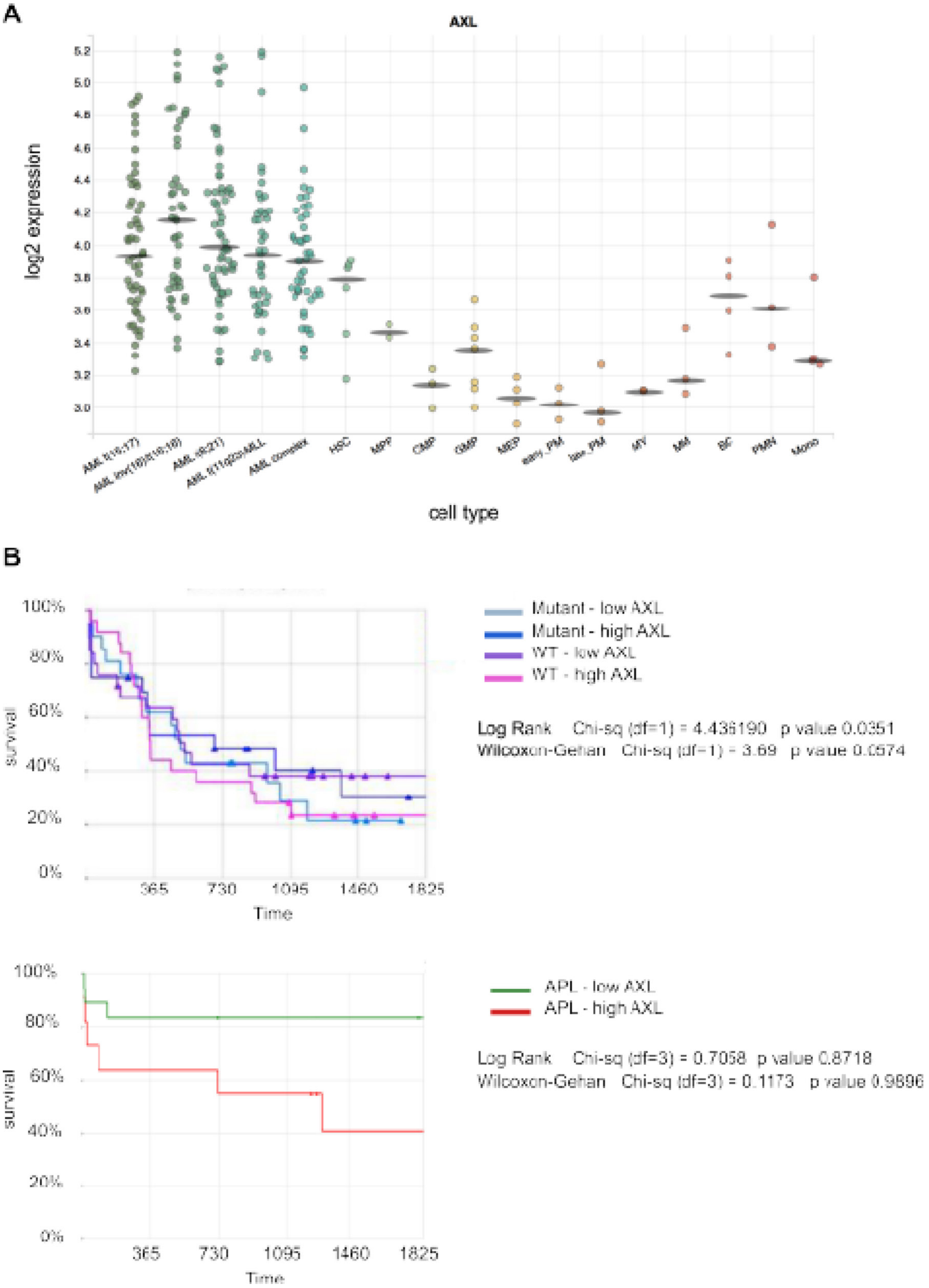


**Supplementary Figure 4: AXL is highly expressed in AML subgroups relative to hematopoietic counterparts.** (**A**) Bloodspot data shows the log2 expression of AXL in AML subtypes (t(15;17), inv(16)/t(16;16), t(8;21) t(11q23)MLL and complex karyotype) and normal hematopoietic counterparts; hematopoietic stem cell (HSC), multipotential progenitor (MPP), common myeloid progenitor (CMP) granulocyte monocyte progenitor (GMP), megakaryocyte-erythroid progenitor (MEP), early promyelocyte (early PM), late promyelocyte (late PM), myelocyte (MY), metamyelocyte (MM), band cell (BC), polymorphonuclear cells (PMN) and monocytes (Mono). (**B**) 5 year survival data of NMP1 mutant or APL patients with either higher or lower than median *AXL* expression.

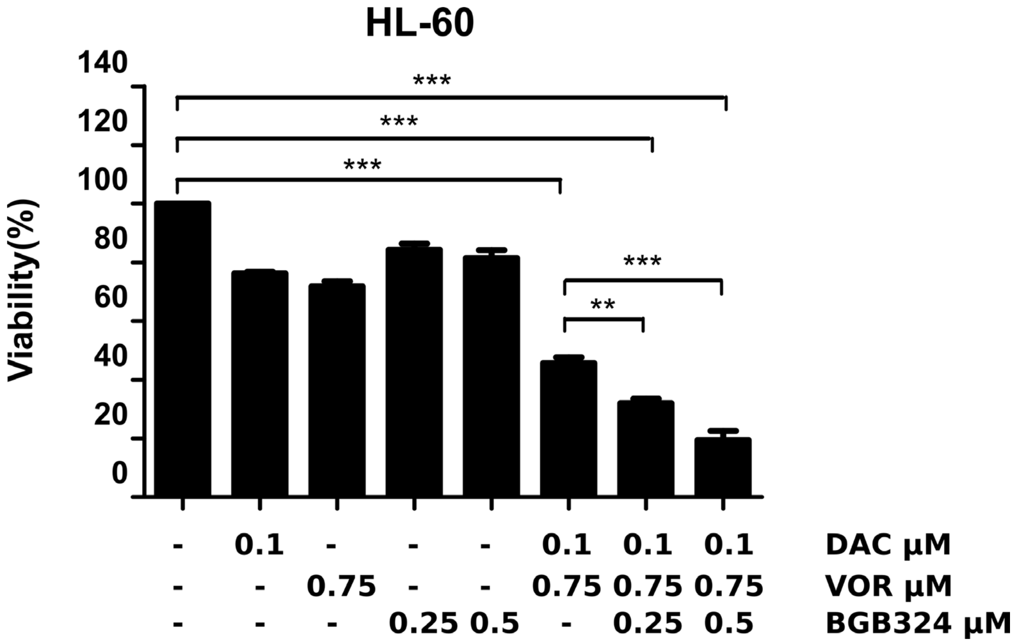


**Supplementary Figure 5: BGB324 reduced the viability of HL-60 cells treated with DV combination in an additive manner.** The viability of HL-60 cells was assessed using CellTitre-Glo® assay and represented as percentage of control. Data represent mean ± SEM; $n$ = 3 (***= $p < 0.001$; **= $p < 0.01$; *= $p < 0.05$).

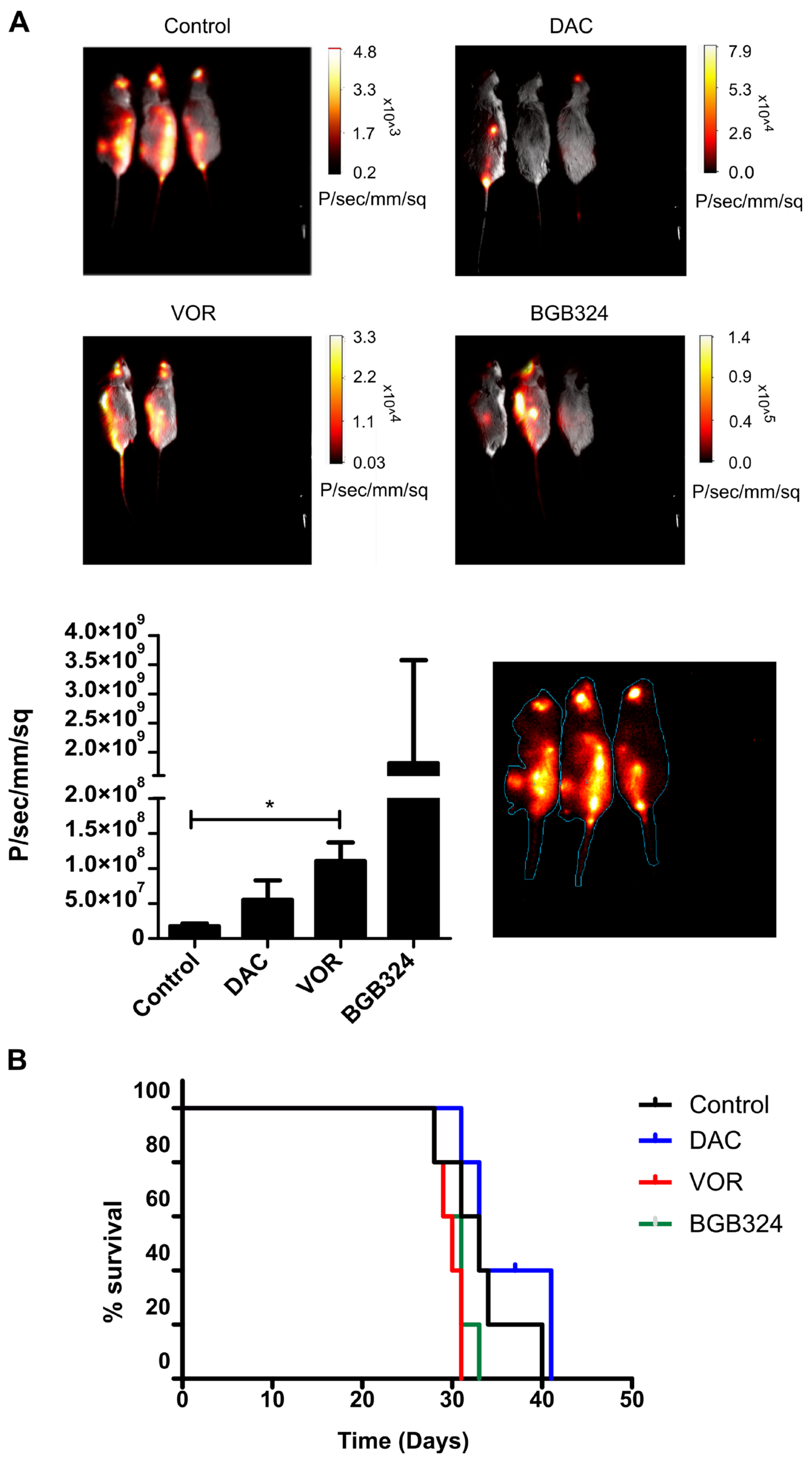


**Supplementary Figure 6: *Ex vivo* single agent treatment had no effect on leukemic burden and survival of mice.** (**A**) OCI-AML3 luciferase cells were treated *in vitro* with vehicle control, or single agent 0.1 µM DAC (72 hours), 0.75 µM VOR (24 hours) or 0.5 µM BGB324 (16 hours). 1.5 × $10^6$ cells were transplanted via tail vein injection into NSG recipient mice ($n$ = 5 per treatment group). Representative images taken at 29 days post transplantation show the disease burden in mice that received control (top left), DAC (top right), VOR (bottom left) and BGB324 (bottom right) treated cells. Average luminescence intensity for each cohort is shown as Photons/sec/mm/sq. A representative image of the ROI is also depicted here. (**B**) Kaplan Meier curves comparing the survival of mice that received control treated OCI-AML3 luciferase cells (black line) to those that received single agent DAC (blue line), VOR (red line) or BGB324 (green line).
